# Oxidative stress gene expression in ulcerative colitis: implications for colon cancer biomarker discovery

**DOI:** 10.1038/s41598-025-05108-8

**Published:** 2025-07-02

**Authors:** Ting Yan, Ting Su, Miaomiao Zhu, Qiyuan Qing, Binjie Huang, Jun Liu, Tenghui Ma

**Affiliations:** 1 Department of Clinical Nutrition, The Sixth Affiliated Hospital of Sun Yat-sen University, Guangzhou, 510000 Guangdong China; 2 Department of Traditional Chinese Medicine, The Sixth Affiliated Hospital of Sun Yat-sen University, Guangzhou, 510000 Guangdong China; 3 Department of Health Information Management, The Sixth Affiliated Hospital of Sun Yat-sen University, Guangzhou, 510000 Guangdong China; 4 Department of Colorectal Surgery, The Sixth Affiliated Hospital of Sun Yat-sen University, Guangzhou, 510000 Guangdong China

**Keywords:** Colorectal cancer, Ulcerative colitis, Bioinformatics, Central gene, Oxidative stress gene, Biomarker, Computational biology and bioinformatics, Oncology

## Abstract

There is a complex interrelationship between colorectal cancer (CRC) and ulcerative colitis (UC). This study aimed to identify key molecules and pathways involved in the co-occurrence of CRC and UC, as well as the role of oxidative stress in disease progression, through bioinformatics analysis of public RNA sequencing databases. We downloaded datasets from public repositories and conducted gene set enrichment analysis (GSEA), screening for oxidative stress-related differentially expressed genes (OXSRDEGs) to evaluate their diagnostic potential. Subsequently, we performed Gene Ontology (GO) analysis and Kyoto encyclopedia of genes and genomes (KEGG) analyses, followed by immune infiltration analysis using the single-sample gene-set enrichment analysis (ssGSEA) and CIBERSORT algorithms. By constructing a multivariate Cox prognostic model using Kaplan–Meier curves and least absolute shrinkage and selection operator (LASSO) regression analysis, we assessed the model’s prognostic capability. Furthermore, we utilized the STRING database and Cytoscape to establish a protein–protein interaction (PPI) network and constructed an mRNA-transcription factor (TF) and mRNA-miRNA interaction networks. The molecular functions and signaling pathways enriched in OXSRDEGs were determined. The robust diagnostic efficacy of OXSRDEGs was verified. This analysis suggests that immune cells may collaborate with OXSRDEGs to impact the onset and progression of diseases. A total of 6 OXSRDEGs with prognostic significance were identified, and the multifactorial Cox regression model constructed demonstrated a strong clinical predictive capacity. The mRNA-transcription factor (TF) and mRNA-miRNA interaction networks revealed that OXSRDEGs are regulated by multiple miRNAs and many transcription factors. Common biomarkers of oxidative stress in the pathogenesis, disease progression, gene expression, and transcription of ulcerative colitis and colorectal cancer have been identified, presenting potential therapeutic targets. The model may be beneficial in prognostic prediction and guiding treatment decisions.

**Supplementary Information:**

The online version contains supplementary material available at 10.1038/s41598-025-05108-8.

## Introduction

Ulcerative colitis (UC) is a chronic inflammatory bowel disease affecting the colon and rectum. It presents significant treatment challenges, such as the an increased risk of malignant transformation, a tendency to recurrence, and a rising incidence rate. The World Health Organization has classified UC as a complex contemporary disease^1^. The onset of UC is believed to be influenced by immune responses, genetic factors, environmental conditions, and intestinal infections, yet the exact causes and underlying mechanisms remain largely unclear^2^. Despite advancements in surgical, radiation, and chemotherapeutic treatments, the prognosis for patients remains poor. Many are diagnosed at an advanced stage, missing the critical window for effective treatment, highlighting the urgent need for the discovery of molecular biomarkers for early detection, prognosis evaluation, and the development of targeted treatments for colorectal cancer (CRC). CRC ranks as one of the most common malignant tumors worldwide. Individuals with UC are at an increased risk of developing CRC, especially colitis-associated colorectal cancer (UC-CRC), which is 2–4 times more likely to occur than in the general population, with a prevalence of approximately 5%^3^. The risk escalates with the duration of the disease. UC-CRC is the most severe complication in UC patients, accounting for about 15% of UC-related mortality^3^.

The permanent stimulation of epithelial proliferation in an inflammatory environment is believed to play an important role in the pathogenesis of UC-CRC in patients with chronic colitis^4,5^. During this process, various factors—such as the excessive production of reactive oxygen and nitrogen species (RONS), the elevated production or activation of crucial arachidonic acid derivatives and cytokines/growth factors (along with their respective signal transduction cascades, e.g., NF-κB), and immune system malfunctions—could all contribute to an elevated risk of cancer and drive the multifaceted development and progression of colorectal cancer^6^. ROS production driven by inflammation leads to oxidative DNA damage, potentially triggering either intrinsic mitochondrial-mediated or extrinsic death receptor-mediated apoptotic pathways, or promoting cellular transformation into tumor cells^3^. Oxidative damage to DNA may result in base modifications, DNA strand breaks, and dysregulation of oncogene expression^7^. Such damage in colonic epithelial cells can arise from genetic instability, mutations in specific genes, and abnormal methylation patterns, thereby facilitating the onset of CRC^3,8^. Consequently, ROS production is considered a key factor in the pathogenesis of chronic UC-CRC^9,10^.

Inflammatory bowel disease (IBD), including UC, has been described as an “oxyradical overload” condition^11–13^. Thus, oxidative stress plays a crucial role in the initiation and progression of both colorectal cancer (CRC) and ulcerative colitis (UC). A deeper understanding of oxidative stress is essential not just for preventing tumor development but also for improving early detection, monitoring CRC progression, identifying novel prognostic biomarkers, and developing innovative prognostic models with advanced in vivo systems.

In this study, we standardized three datasets (TCGA-COADREAD, GSE74602, and GSE4183) and analyzed differentially expressed genes (DEGs), integrating multi-omics data to enhance reliability. Using ssGSEA and CIBERSORT algorithms for immune cell infiltration analysis, we identified significant differences between disease and control groups. Spearman correlation analysis further revealed relationships between immune cell infiltration and 12 oxidative stress-related DEGs (OXSRDEGs).

Our systematic analyses uncovered the expression profiles and diagnostic potential (via ROC curves) of these genes across datasets. We also established their prognostic relevance (through Cox modeling), particularly highlighting *NFE2L3*'s critical role in the NF-κB pathway. We developed, validated, and confirmed a multivariate Cox model incorporating six prognostic OXSRDEGs (*CXCL11, MMP10, MMP3, NFE2L3, RNASE1, TIMP1*) using Kaplan–Meier survival analysis and LASSO regression. The model’s clinical utility was assessed via nomogram and decision curve analyses.

Using STRING and Cytoscape, we constructed a PPI network, while CHIPBase and miRDB databases predicted mRNA-TF and mRNA-miRNA interaction networks, revealing key genes and their regulatory mechanisms. Comprehensive analysis of TCGA-COADREAD clinical data evaluated OXSRDEG expression patterns and prognostic value in CRC patients. Furthermore, by examining gene expression-immune cell infiltration relationships, we identified immune regulatory roles of *CXCL11, MGP*, and *PPARGC1A*.

These findings provide a crucial theoretical foundation and robust dataset for exploring molecular mechanisms, early diagnostic markers, and therapeutic targets in colorectal cancer and ulcerative colitis.

## Materials and methods

### Data download

The TCGAbiolinks package^14^ facilitated the download of the colorectal cancer dataset (TCGA-COADREAD) from The Cancer Genome Atlas (TCGA), which served as the test set for our analysis. We excluded samples that lacked clinical survival information, resulting in a dataset comprising 695 CRC samples with clinical Overall Survival (OS) information, including 51 control samples and 644 COADREAD samples. The corresponding clinical data were sourced from the UCSC Xena database^15^.

For the acquisition of expression profile datasets for CRC and UC patients, we employed the R package GEOquery^16^. Specifically, the CRC patient dataset (GSE74602) and the UC patient dataset (GSE4183)^17^ were downloaded from the GEO (Gene Expression Omnibus) database, with both datasets specific to Homo sapiens. The GSE74602 dataset consisted of 30 tumor samples from CRC patients (group: COADREAD) and 30 normal colon tissue samples (group: Control).

The GSE4183 dataset included 9 colon samples from UC patients and 8 frozen colon tissue samples from healthy controls, amounting to a total of 17 samples.

The data platforms for these datasets were distinct: the GSE74602 dataset utilized the GPL6104 Illumina humanRef-8 v2.0 expression BeadChip, whereas the GSE4183 dataset was based on the GPL570 [HG-U133_Plus_2] Affymetrix Human Genome U133 Plus 2.0 Array. The probe names in both datasets were annotated using the respective chip GPL platform files, with detailed information presented in Table 1.

**Table 1:** Data set information list.

	TCGA-COADREAD	GSE74602	GSE4183
Platforms	–	GPL6104 Illumina humanRef-8 v2.0 expression BeadChip	GPL570 [HG-U133_Plus_2] Affymetrix Human Genome U133 Plus 2.0 Array
Experiment type	–	Expression profiling by array	Expression profiling by array
Organism	Homo sapiens	Homo sapiens	Homo sapiens
Tissue source	Colon	Colon	colon
Group	COADREAD/Control	COADREAD/Control	UC/Control
References (title)	–	–	Inflammation, adenoma and cancer: objective classification of colon biopsy specimens with gene expression signature

*TCGA* the cancer genome atlas, *COADREAD* colorectal carcinoma, *UC* ulcerative colitis.

To identify oxidative stress-related genes (OXSRGs), we consulted the GeneCards database^18^, utilizing “Oxidative stress” as the search criterion. This approach yielded a comprehensive list of genes associated with oxidative stress. After filtering to retain only those that are protein-coding, a total of 7410 OXSRGs were identified, indicating the variety of potential targets for further investigation into their roles in UC and CRC pathogenesis.

### Data processing and differential expression analysis

The datasets TCGA-COADREAD, GSE74602, and GSE4183 were initially normalized using the limma package^19^, followed by a comparative evaluation through boxplots of the expression matrix pre- and post-normalization.

Subsequent differential analysis among the groups within these datasets was conducted using the limma package to identify differentially expressed genes (DEGs) across the datasets. The DEGs were then refined based on the criteria of |logFC|> 1 and P.adj < 0.05, leading to the identification of a subset of DEGs for further analysis. These results were visually represented using the ggplot2 package in R, classifying genes with logFC > 1 and P.adj < 0.05 as up-regulated DEGs, and those with logFC < -1 and P.adj < 0.05 as down-regulated DEGs.

To discover OXSRDEGs that are differentially expressed in both the COADREAD and UC datasets, the subset of DEGs was intersected with OXSRGs, resulting in the identification of OXSRDEGs. Following this, a univariate COX analysis was employed to screen the OXSRDEGs, with only those satisfying *p* < 0.05 being retained for further study.

### Gene function enrichment analysis (GO) and pathway enrichment (KEGG) analysis

Gene Ontology (GO) analysis^20^ serves as a foundational approach for large-scale functional enrichment studies, spanning three domains: Biological Process (BP), Molecular Function (MF), and Cellular Component (CC). The Kyoto Encyclopedia of Genes and Genomes (KEGG)^21^, a comprehensive database, catalogues information on genomes, biological pathways, diseases, and pharmaceuticals. Utilizing the R package clusterProfile^22^, GO and KEGG annotation analyses were performed on OXSRDEGs, with selection criteria set at P.adj < 0.05 and an FDR value (q.value) < 0.05, employing the Benjamini-Hochberg (BH) method for P-value correction.

### GSEA

Gene Set Enrichment Analysis (GSEA)^23^ employs a computational strategy to assess if a pre-defined gene set exhibits statistically significant differences in two distinct biological states, frequently applied to deduce variations in pathway and biological process activities within gene expression datasets. Initially, genes were categorized into positive and negative cohorts based on logFC values derived from differential analysis, followed by enrichment analysis on these groups using clusterProfiler. GSEA parameters included a seed of 2022, 1000 calculations, gene set size limits ranging from 10 to 500, and the BH correction method for P-values. The reference gene set "c2.cp.all.v2022.1.Hs.symbols.gmt All Canonical Pathways" was sourced from the MSigDB database^24^, with significant enrichment criteria set at P.adj < 0.05 and FDR value (q.value) < 0.05.

### Identification and correlation analysis of immune infiltration cells in TCGA-COADREAD and GSE4183 datasets

Identification and Correlation Analysis of Immune Infiltration Cells in the TCGA-COADREAD and GSE4183 Datasets utilized SSGSEA to compute enrichment scores reflecting the extent of immune cell infiltration, annotated across both datasets^25,26^. Boxplots illustrated the variance in immune cell infiltration abundance. Correlations among immune cells within the datasets were determined using the Spearman statistical method and depicted through the ggplot2 package in R. Additionally, the correlation between significantly varied immune cells and OXSRDEGs was analyzed based on the OXSRDEGs expression matrix and visualized via ggplot2.

CIBERSORT^27^, an algorithm based on linear support vector regression, deconvolutes transcriptome expression matrices to infer the composition and quantity of immune cells within mixed cell populations. The gene expression matrix data was processed through CIBERSORT, alongside the LM22 signature gene matrix, selecting data with immune cell enrichment scores above zero. This process yielded detailed outcomes of immune cell infiltration abundance, visually represented in a bar stacked chart. Variations in immune cell infiltration abundance across different groups were evaluated using the Wilcoxon rank sum test, with boxplots displaying the results. The correlation among immune cells was also computed using the Spearman algorithm and depicted through ggplot2. Lastly, the relationship between significantly diverse immune cells and OXSRDEGs across groups was analyzed based on the OXSRDEGs expression matrix and illustrated using ggplot2.

### Construction of a multivariate cox prognostic model

The survival package was utilized to conduct Kaplan–Meier (KM) curve analysis, categorizing OXSRDEGs expression values into High and Low groups according to the median, to examine the disparities in overall survival (OS) between the high and low expression groups of OXSRDEGs, as well as between the high and low-risk groups of the TCGA-COADREAD dataset. Only OXSRDEGs demonstrating a p-value of less than 0.05 were selected for further analysis.

Subsequently, the glmnet package^28^ was employed, with parameters set.seed (500) and family = "binomial," to carry out LASSO regression analysis^29^ on OXSRDEGs. The purpose of incorporating a running cycle of 500 was to mitigate the risk of overfitting. LASSO regression, which stands for Least Absolute Shrinkage and Selection Operator, enhances the process of linear regression by introducing a penalty term (lambda × the absolute value of the coefficient), effectively diminishing the likelihood of model overfitting and concurrently boosting the model’s ability to adapt to new data.

$$\text{risk}Score = \sum_{i}Coefficient \left({ERSRDEGs}_{i}\right)*mRNA Expression ({ERSRDEGs}_{i})$$

The OXSRDEGs identified through LASSO analysis were employed as prognostic OXSRDEGs for the subsequent development of a multivariate Cox prognostic model.

All prognostic OXSRDEGs were subjected to multivariate Cox regression analysis, leading to the establishment of a multivariate Cox prognostic model. The R package “rms” facilitated the creation of a nomogram chart^30^, which is depicted on a plane Cartesian coordinate system and illustrates the functional relationship among various independent variables through a series of non-overlapping segments. This approach is rooted in multivariate regression analysis. It involves assigning scores to the variables within the multivariate regression model on a specific scale, and then aggregating these scores to forecast the likelihood of an event’s occurrence.

In the final step, the accuracy and discriminative power of the multivariate Cox prognostic model’s nomogram chart were assessed using a calibration curve. The calibration curve (Calibration) chart^31^ represents a graphical comparison between the actual probabilities and those predicted by the model under various conditions, serving as a measure of the model’s predictive accuracy. Decision Curve Analysis (DCA)^32^ offers a straightforward technique for evaluating clinical prediction models, diagnostic tests, and molecular markers. Consequently, the DCA chart was employed to examine the model’s accuracy and discriminative capability, with the R package “ggDCA” being utilized to produce the DCA chart, thereby assessing the predictive performance of the multivariate Cox prognostic model.

### Construction of protein–protein interaction network (PPI Network)

The PPI network is constituted by individual proteins that interact with one another, playing vital roles in a multitude of biological processes including signal transduction, regulation of gene expression, metabolism, and regulation of the cell cycle. The STRING database serves as a comprehensive resource for exploring both known and predicted protein interactions. In this research, the STRING database^33^ was employed to assemble the PPI network of Prognostic OXSRDEGs, with the network model being depicted through the use of Cytoscape.

### Construction of mRNA-TF, mRNA-miRNA interaction networks

The CHIPBase database (version 3.0) (https://rna.sysu.edu.cn/chipbase/) extracts thousands of binding motif matrices along with their binding sites from ChIP-seq data pertaining to DNA-binding proteins, in addition to forecasting hundreds of thousands of transcriptional regulatory connections between transcription factors (TF) and genes. The hTFtarget database^34^ (http://bioinfo.life.hust.edu.cn/hTFtarget.) is an exhaustive repository that houses data on human TFs and their target genes. Searches for TFs binding to Prognostic OXSRDEGs were conducted utilizing both the CHIPBase and hTFtarget databases, with the findings being graphically represented in Cytoscape software.

The ENCORI database^35^ (https://starbase.sysu.edu.cn/), a component of the starBase platform version 3.0, specializes in the interactions between microRNAs and mRNAs, drawing on data mining from CLIP-seq and degradation group sequencing. It offers a range of graphical interfaces for the investigation of miRNA targets. The ENCORI database was utilized to forecast the miRNAs that interact with Prognostic OXSRDEGs, applying a screening criterion of pancancerNum > 5 to identify interaction relationships. The mRNA-miRNA interaction network was subsequently visualized using Cytoscape.

### Statistical analysis

**Fig. 1:**
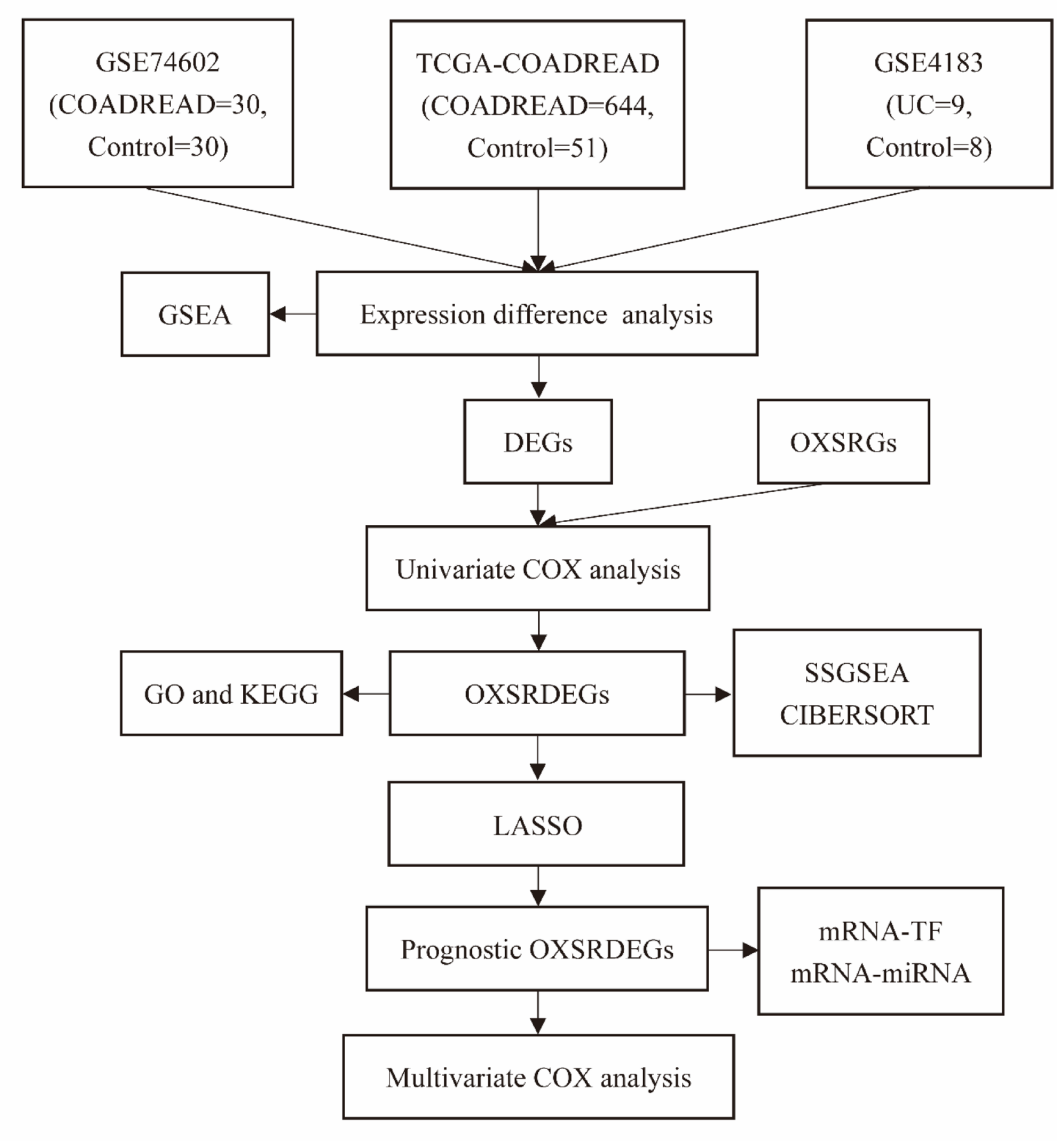
Schematic diagram of the study. The abbreviations used throughout the diagram include TCGA (The Cancer Genome Atlas), COADREAD (Colorectal Carcinoma), UC (Ulcerative Colitis), GSEA (Gene Set Enrichment Analysis), DEGs (Differentially Expressed Genes), OXSRGs (Oxidative Stress-Related Genes), OXSRDEGs (Oxidative Stress-Related Differentially Expressed Genes), GO (Gene Ontology), KEGG (Kyoto Encyclopedia of Genes and Genomes), SSGSEA (Single-Sample Gene-Set Enrichment Analysis), LASSO (Least Absolute Shrinkage and Selection Operator), PPI Network (Protein–Protein Interaction Network), and TF (Transcription Factors).

All data processing and analysis in this study were conducted using R software (Version 4.2.2), with continuous variables described as mean ± standard deviation. For comparisons between two groups, the Wilcoxon rank sum test was employed. When comparing three or more groups, the Kruskal–Wallis test was utilized. Unless otherwise stated, Spearman correlation analysis was applied to determine the correlation coefficients among various molecules. Statistical significance was assessed bilaterally for all P-values, with a threshold of P < 0.05 indicating statistical significance (Fig. 1).

## Results

### Colorectal cancer dataset processing and differential expression analysis

**Fig. 2:**
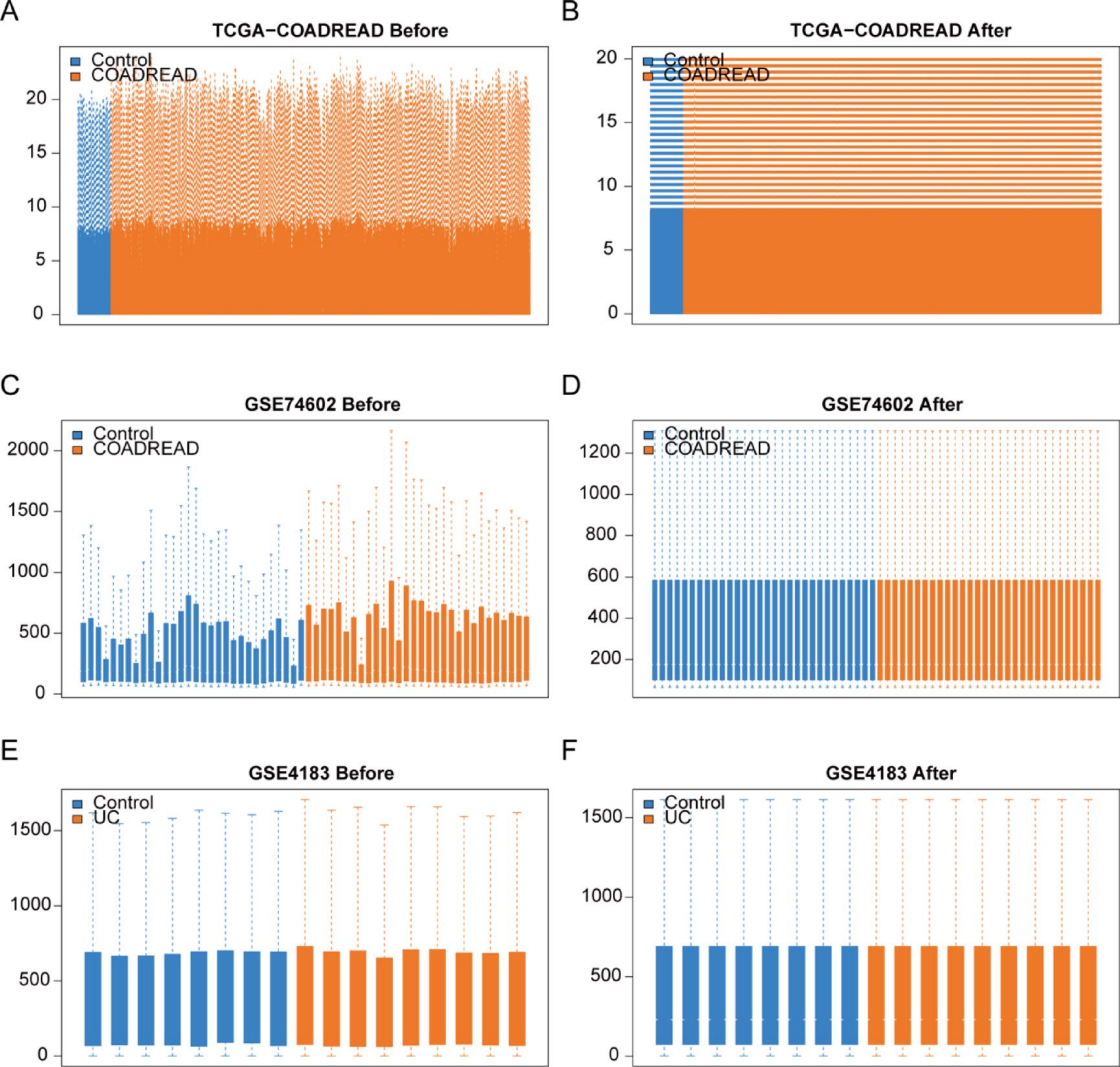
Standardization processing of three datasets. (**A**, **B**) Boxplot illustrating the TCGA-COADREAD dataset before (**A**) and after (**B**) standardization processing. (**C**, **D**) Boxplot depicting the GSE74602 dataset before (**C**) and after (**D**) standardization processing. (**E**, **F**) Boxplot presenting the GSE4183 dataset before (**E**) and after (**F**) standardization processing. Notably, TCGA refers to The Cancer Genome Atlas, COADREAD signifies Colorectal carcinoma, and UC represents Ulcerative colitis.

Firstly, we normalized the three datasets, namely TCGA-COADREAD, GSE74602, and GSE4183, utilizing the normalizeBetweenArrays function from the R package limma (Fig. 2A–F). The TCGA-COADREAD dataset is comprised of 695 samples, encompassing 51 control samples and 644 CRC samples (Fig. 2A,B). The GSE74602 dataset consists of a total of 60 samples, with 30 control samples (group: Control) and 30 CRC samples (group: COADREAD) (Fig. 2C,D). Similarly, the GSE4183 dataset encompasses 17 samples, including 8 control samples (group: Control) and 9 ulcerative colitis samples (group: UC) (Fig. 2E,F). Illustrated in Fig. 2A–F, the expression profile data of the TCGA-COADREAD, GSE74602, and GSE4183 datasets exhibit a tendency towards consistency post-normalization processing.

**Fig. 3:**
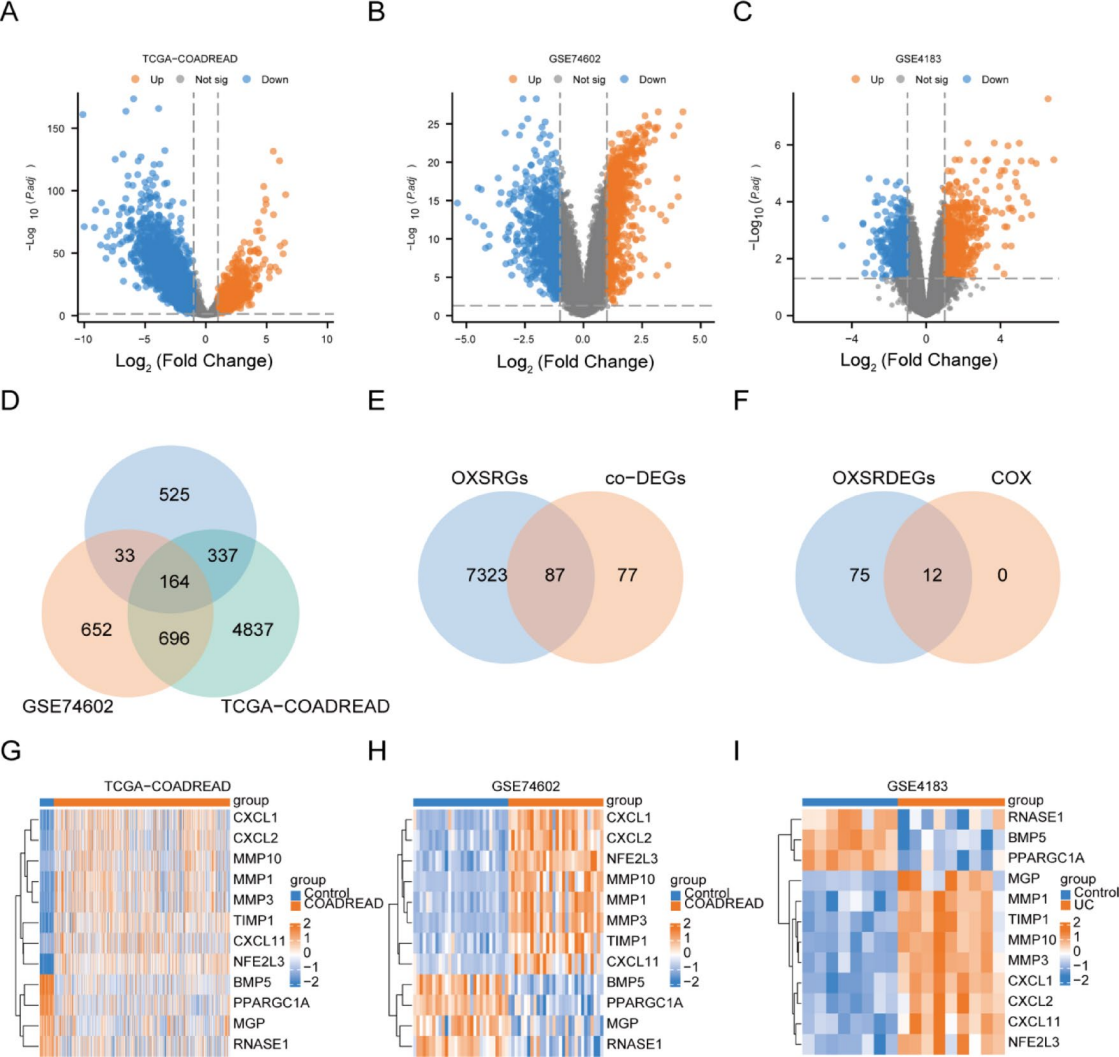
Differential expression analysis of three datasets. (**A**–**C**) Volcano plots illustrating the results of the differential analysis between disease and control groups in the TCGA-COADREAD dataset (**A**), GSE74602 dataset (**B**), and GSE4183 dataset (**C**). (**D**) Venn diagram showcasing the overlap of differentially expressed genes (DEGs) in the three datasets. (**E**) Venn diagram highlighting the common differentially expressed genes (co-DEGs) and oxidative stress-related genes (OXSRGs). (**F**) Venn diagram presenting the intersection of COX univariate screening results and OXSRDEGs. (**G**–**I**) Heatmaps depicting the expression of OXSRDEGs in disease and control groups for TCGA-COADREAD (**G**), GSE74602 (**H**), and GSE4183 (**I**). TCGA, The Cancer Genome Atlas; UC, Ulcerative colitis; COADREAD, Colorectal carcinoma; DEGs, differentially expressed genes; co-DEGs, common DEG; OXSRGs, Oxidative stress-related genes; OXSRDEGs, Oxidative stress-related differentially expressed genes.

To examine the variations in gene expression across different groups within the three datasets, Differentially Expressed Genes (DEGs) were identified using the limma package for each dataset. The outcomes are detailed below: In the TCGA-COADREAD dataset, 6034 genes satisfy the criteria |logFC|> 1 and P.adj < 0.05. Within this set, 824 genes exhibit high expression in the COADREAD group (with lower expression in the Control group, indicated by a positive logFC, signifying up-regulated genes), while 5210 genes showcase low expression in the COADREAD group (with higher expression in the Control group, denoted by a negative logFC, indicating down-regulated genes). The results of the differential analysis for the TCGA-COADREAD dataset were visually represented through a volcano plot (Fig. 3A).

Within the GSE74602 dataset, 1545 genes fulfill the criteria |logFC|> 1 and P.adj < 0.05. Among them, 730 genes exhibit heightened expression in the COADREAD group (with lower expression in the Control group, reflected by a positive logFC denoting up-regulated genes), while 815 genes display reduced expression in the COADREAD group (with higher expression in the Control group, indicated by a negative logFC, representing down-regulated genes). The outcomes of the differential analysis for the GSE74602 dataset were visually depicted through a volcano plot (Fig. 3B).

As for the GSE4183 dataset, 1059 genes meet |logFC|> 1 and P.adj < 0.05. Within this set, 640 genes demonstrate elevated expression in the UC group (with lower expression in the Control group, logFC is positive, indicating up-regulated genes), while 419 genes show diminished expression in the UC group (with higher expression in the Control group, logFC is negative, signifying down-regulated genes). The differential analysis results for the GSE4183 dataset were illustrated as a volcano plot (Fig. 3C).

To identify Overlapping Differentially Expressed Genes (OXSRDEGs), the intersection of all DEGs meeting |logFC|> 1 and P.adj < 0.05 across the three datasets was determined (Fig. 3D). This yielded 164 genes exhibiting expression disparities among all three datasets (common DEGs or co-DEGs), with specific names listed in Table 2. Subsequently, the intersection of co-DEGs and oxidative stress-related genes (OXSRGs) was established (Fig. 3E), resulting in 87 OXSRDEGs, with specific names detailed in Table 2.

**Table 2:** co-DEGs list and OXSRDEGs list.

co-DEGs	co-DEGs	co-DEGs	co-DEGs
DUOXA2	PMM1	MYEOV	NEU4
LCN2	PHLDA1	SLC39A5	SLCO4A1
REG1A	CFD	LRRN2	SLC30A10
MMP3	PCK1	TSPAN7	CD160
SLC6A14	FJX1	DUSP4	CA2
CXCL1	OAF	SLC26A2	PBLD
CXCL6	SLC36A1	AMPD1	ZG16
MMP10	LRP8	NPY	RDH5
ABCA8	TRPM6	CA1	UGT2A3
ITPKA	PPARGC1A	CAPN9	PYY
CXCL5	SLC22A5	CKB	ADAMTS1
IFITM1	SLC17A4	GHR	LRRC19
TIMP1	AQP9	KRT6B	OSM
REG1B	CXCL10	TEAD4	CLDN2
ENTPD5	CLCN2	PIGZ	TNFAIP8L3
PLP1	PSAT1	AQP8	XDH
MMP28	KLK11	FAP	F13A1
ZNF91	VIPR1	HSD11B2	MMRN1
SLC16A9	PLEKHG6	CDH3	ADH1C
PDPN	LAMA1	EDN3	PPP1R16B
GPM6B	SPON1	STC1	IGSF9
STOM	CTHRC1	HMGCS2	CORO1A
SLC7A5	FRMD1	LRRC31	TTLL6
MAMDC2	ABCG2	RAVER2	PCSK9
MMP7	NFE2L3	COL12A1	CHGA
SERPINE1	SELENBP1	HCLS1	CYP4F12
CXCL11	ABCB1	MAOA	SCD
SGK2	ISX	SLAMF7	TEX11
PLAU	CAMK2N1	PDE6A	RNASE1
MMP1	SERPINB5	LAG3	GUCA2B
PLEKHO1	SLC25A34	BEST2	DEFA6
CAPN13	RGS5	SLC26A3	LDHD
EPB41L4B	NDRG2	PNPLA7	CD79A
S100A11	MYOT	MGP	MT1M
TCN1	CDKN2B	TUBAL3	MS4A12
BMP5	CEP55	TCEA3	MT1F
CLDN8	THBS2	GUCA2A	BEST4
CXCL2	CLDN1	SPINK4	SPP1
FOXQ1	RHOU	PRR7	SCN9A
CDC25B	SLC4A4	TNFRSF12A	SCGB2A1
PRDX6	RAPGEFL1	S100P	CA7

OXSRDEGs	OXSRDEGs	OXSRDEGs	
XDH	AQP8	REG1A	
PRDX6	CXCL6	STOM	
PPARGC1A	CDH3	CLDN1	
SERPINE1	SELENBP1	CD160	
MAOA	CFD	TEAD4	
MMP1	FAP	S100P	
TIMP1	TRPM6	NFE2L3	
PLAU	ADAMTS1	LRRN2	
MMP3	CXCL10	PIGZ	
SPP1	CA2	ZG16	
CDKN2B	ITPKA	CA7	
CXCL1	LAMA1	TCEA3	
LCN2	STC1	GUCA2A	
NPY	SLC7A5	F13A1	
PCK1	SLC22A5	MGP	
ABCB1	DUSP4	ADH1C	
SERPINB5	SGK2	GHR	
CXCL2	SCD	HMGCS2	
TNFRSF12A	PCSK9	THBS2	
CAPN9	CXCL11	CLCN2	
MMP7	CXCL5	LRP8	
ABCG2	NDRG2	CA1	
MT1F	RNASE1	PSAT1	
CKB	MMP10	BMP5	
MYOT	HSD11B2	CTHRC1	
SLC30A10	CORO1A	CLDN2	
CD79A	OSM	TUBAL3	
CHGA	S100A11	CLDN8	
CDC25B	CEP55	MT1M	

*co-DEGs* common DEG.

*OXSRDEGs* oxidative stress related differentially expressed genes.

A univariate COX analysis was executed on the 87 OXSRDEGs, employing a screening criterion of p < 0.05, resulting in the retention of 12 OXSRDEGs (Fig. 3F). These retained genes are *BMP5, CXCL1, CXCL11, CXCL2, MGP, MMP1, MMP10, MMP3, NFE2L3, PPARGC1A, RNASE1*, and *TIMP1*.

Heatmaps were generated to visualize the expression patterns of the 12 OXSRDEGs in the TCGA-COADREAD dataset (Fig. 3G), GSE74602 dataset (Fig. 3H), and GSE4183 dataset (Fig. 3I). Notably, the expression of these 12 OXSRDEGs exhibits significant differences among groups in the three datasets.

### Functional enrichment analysis (GO) and pathway enrichment (KEGG) Analysis of OXSRDEGs

**Fig. 4:**
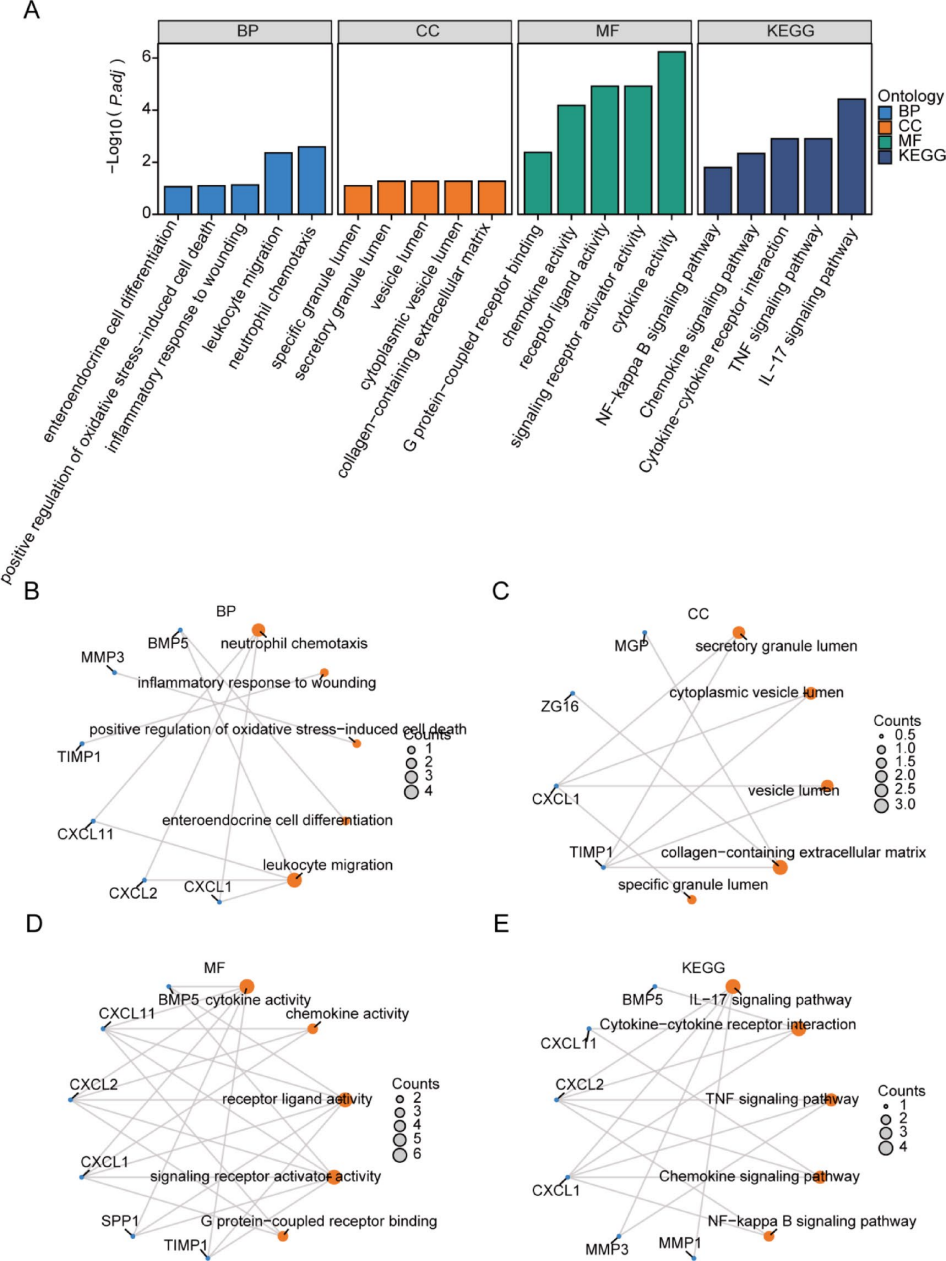
Functional enrichment analysis of OXSRDEGs (GO). The bar chart illustrates the outcomes of (**A**). OxsrDEGS GO enrichment analysis and KEGG pathway enrichment analysis. The loop network diagram, denoted by BP (**B**), CC (**C**), MF (**D**), and KEGG (**E**), showcases the GO functional enrichment analysis results for OXSRDEGs (Oxidative stress-related differentially expressed genes). In the bar chart (**A**), GO terms or KEGG terms are depicted on the horizontal axis, with the bar height corresponding to the P.AJ value of the respective terms. Within the network diagram (**B**, **C**, **D**, **E**), specific genes are represented by blue dots, while specific pathways are denoted by orange dots. OXSRDEGs refer to Oxidative stress-related differentially expressed genes, and the abbreviations GO, BP, CC, MF, and KEGG stand for Gene Ontology, biological process, cellular component, molecular function, and Kyoto Encyclopedia of Genes and Genomes, respectively. The screening criteria applied for GO/KEGG enrichment items were a P.DJ value less than 0.05 and an FDR value (q.vue) less than 0.05.

To examine the correlation between the 12 Oxidative Stress-Related Differentially Expressed Genes (OXSRDEGs) in the TCGA-COADREAD dataset and Colorectal Carcinoma (CRC or UC) concerning biological processes, molecular functions, cellular components, and biological pathways, Gene Ontology (GO) gene function enrichment and Kyoto Encyclopedia of Genes and Genomes (KEGG) pathway enrichment analyses were initially conducted on the 12 OXSRDEGs (Table 3). The selection criteria for enriched items were set at P.adj < 0.05 and False Discovery Rate (FDR) value (q.value) < 0.05. The findings revealed that the 12 OXSRDEGs were predominantly enriched in various biological processes, including enteroendocrine cell differentiation, positive regulation of oxidative stress-induced cell death, inflammatory response to wounding, leukocyte migration, and neutrophil chemotaxis. Additionally, they exhibited enrichment in cellular components such as secretory granule lumen, cytoplasmic vesicle lumen, vesicle lumen, collagen-containing extracellular matrix, and specific granule lumen. Furthermore, molecular functions like cytokine activity, chemokine activity, receptor ligand activity, signaling receptor activator activity, and G protein-coupled receptor binding were identified. The OXSRDEGs were also significantly enriched in KEGG pathways, including the IL-17 signaling pathway, Cytokine-cytokine receptor interaction, TNF signaling pathway, Chemokine signaling pathway, and NF-kappa B signaling pathway (Fig. 4A). The primary biological processes (Fig. 4B), cellular components (Fig. 4C), molecular functions (Fig. 4D), and KEGG pathways (Fig. 4E) enriched by the 12 OXSRDEGs were visually represented in a circular network diagram.

**Table 3:** GO and KEGG enrichment analysis results of OXSRDEGs.

Ontology	ID	Description	p.adjust	qvalue
BP	GO:0,030,593	Neutrophil chemotaxis	0.001327	0.000755
BP	GO:0,090,594	Inflammatory response to wounding	0.067463	0.03839
BP	GO:1,903,209	Positive regulation of oxidative stress-induced cell death	0.069035	0.039285
BP	GO:0,035,883	Enteroendocrine cell differentiation	0.074564	0.042431
BP	GO:0,050,900	Leukocyte migration	0.001889	0.001075
CC	GO:0,034,774	Secretory granule lumen	0.082046	0.046061
CC	GO:0,060,205	Cytoplasmic vesicle lumen	0.082046	0.046061
CC	GO:0,031,983	Vesicle lumen	0.082046	0.046061
CC	GO:0,062,023	Collagen-containing extracellular matrix	0.086317	0.048458
CC	GO:0,035,580	Specific granule lumen	0.086317	0.048458
MF	GO:0,005,125	cytokine activity	5.62E−06	3.15E−06
MF	GO:0,008,009	chemokine activity	6.01E−05	3.36E−05
MF	GO:0,048,018	receptor ligand activity	8.86E−05	4.96E−05
MF	GO:0,030,546	signaling receptor activator activity	8.86E−05	4.96E−05
MF	GO:0,001,664	G protein-coupled receptor binding	0.00252	0.001411
KEGG	hsa04657	IL-17 signaling pathway	1.84E−05	9.97E−06
KEGG	hsa04060	Cytokine-cytokine receptor interaction	0.000688	0.000373
KEGG	hsa04668	TNF signaling pathway	0.000736	0.000399
KEGG	hsa04062	Chemokine signaling pathway	0.002711	0.00147
KEGG	hsa04064	NF-kappa B signaling pathway	0.011772	0.006384

*GO* gene ontology, *BP* biological process, *CC* cellular component, *MF* molecular function, *OXSRDEGs* oxidative stress related differentially expressed genes.

### GSEA of TCGA-COADREAD and GSE4183

**Fig. 5:**
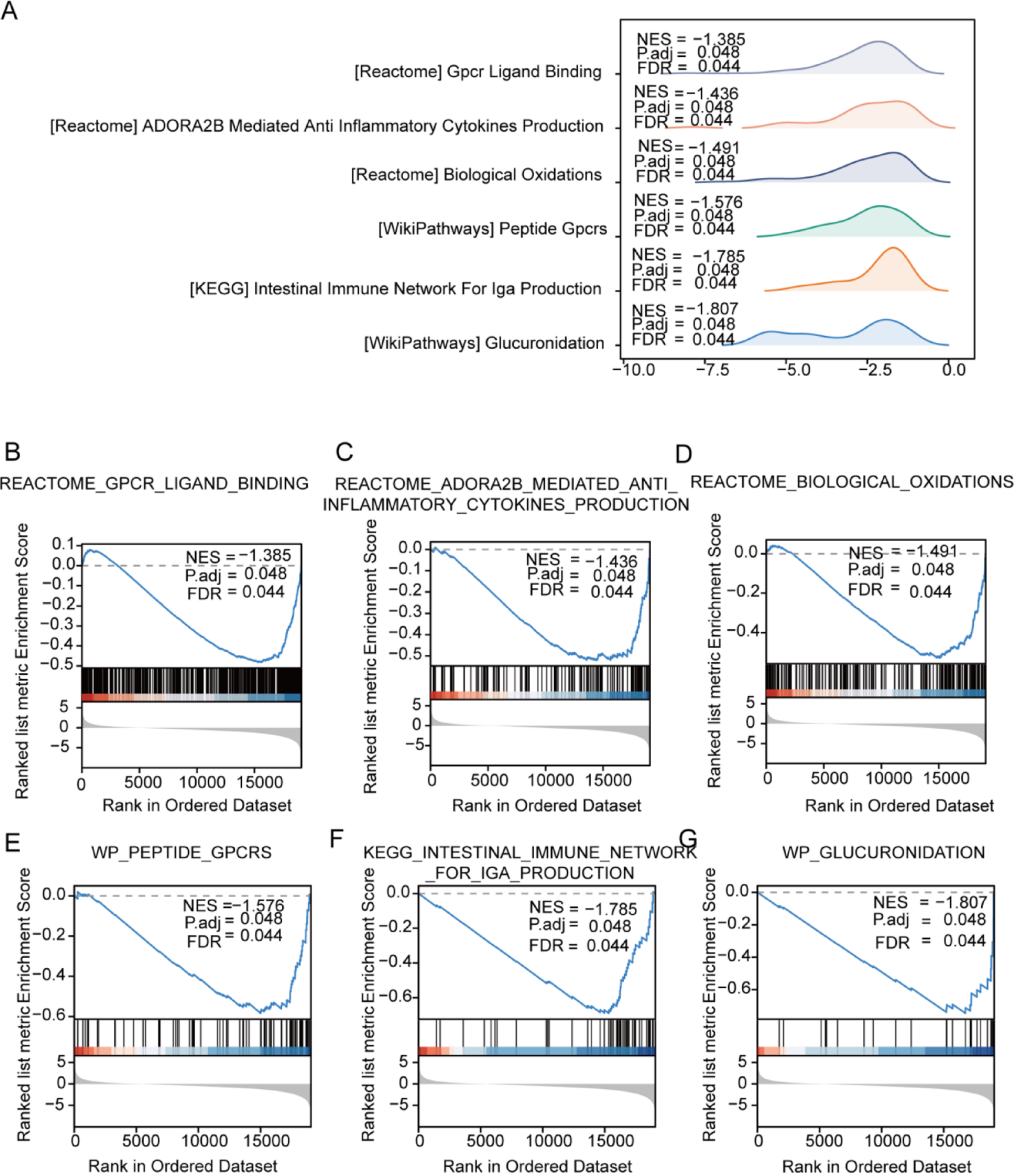
GSEA Enrichment Analysis of TCGA-COADREAD Dataset. (**A**) Illustrates the primary six biological features of gene enrichment between groups in the TCGA-COADREAD dataset. (**B**–**G**) Highlight the significant enrichment of genes in the TCGA-COADREAD dataset within WP_GLUCURONIDATION (**B**), KEGG_INTESTINAL_IMMUNE_NETWORK_FOR_IGA_PRODUCTION (**C**), WP_PEPTIDE_GPCRS (**D**), REACTOME_BIOLOGICAL_OXIDATIONS (**E**), REACTOME_ADORA2B_MEDIATED_ANTI_INFLAMMATORY_CYTOKINES_PRODUCTION (**F**), and REACTOME_GPCR_LIGAND_BINDING (**G**). GSEA, Gene Set Enrichment Analysis; TCGA, The Cancer Genome Atlas; COADREAD, Colorectal Carcinoma. The significant enrichment criteria for GSEA were P.adj < 0.05 and FDR value (q.value) < 0.05.

In the examination of TCGA-COADREAD and GSE4183 datasets (2.3 GSEA), the impact of gene expression variations in colorectal cancer (CRC) groups was assessed. GSEA enrichment analysis was conducted to examine the connection between the expression levels of all genes within these groups and their involvement in biological processes, affected cellular components, and molecular functions. The criteria for significant enrichment were set at P.adj < 0.05 and FDR value (q.value) < 0.05. The findings revealed notable enrichment of genes between the two CRC groups in pathways such as WP_GLUCURONIDATION (Fig. 5B), KEGG_INTESTINAL_IMMUNE_NETWORK_FOR_IGA_PRODUCTION (Fig. 5C), WP_PEPTIDE_GPCRS (Fig. 5D), REACTOME_BIOLOGICAL_OXIDATIONS (Fig. 5E), REACTOME_ADORA2B_MEDIATED_ANTI_INFLAMMATORY_CYTOKINES_PRODUCTION (Fig. 5F), and REACTOME_GPCR_LIGAND_BINDING (Fig. 5G) (refer to Fig. 5B–G and Table 4). The GSEA results were visually presented through a landscape plot (Fig. 5A).

**Table 4:** GSEA enrichment analysis.

ID	Enrichment score	NES	p.adjust	qvalue
GSEA enrichment analysis results of TCGA-COADREAD dataset				
WP_GLUCURONIDATION	− 0.74493	− 1.80676	0.04803	0.043893
KEGG_INTESTINAL_IMMUNE_NETWORK_FOR_IGA_PRODUCTION	− 0.68942	− 1.78536	0.04803	0.043893
WP_PEPTIDE_GPCRS	− 0.58377	− 1.57572	0.04803	0.043893
REACTOME_BIOLOGICAL_OXIDATIONS	− 0.52996	− 1.49067	0.04803	0.043893
REACTOME_ADORA2B_MEDIATED_ANTI_INFLAMMATORY_CYTOKINES_PRODUCTION	− 0.52039	− 1.43622	0.04803	0.043893
REACTOME_GPCR_LIGAND_BINDING	− 0.48201	− 1.38481	0.04803	0.043893
GSEA enrichment analysis results of GSE4183 dataset				
WP_IL18_SIGNALING_PATHWAY	0.574568	2.353922	0.019147	0.01387
WP_INFLAMMATORY_BOWEL_DISEASE_SIGNALING	0.693156	2.161687	0.019147	0.01387
WP_IL4_SIGNALING_PATHWAY	0.633035	2.063739	0.019147	0.01387
WP_CYTOKINES_AND_INFLAMMATORY_RESPONSE	0.722968	2.012929	0.019147	0.01387
WP_IL6_SIGNALING_PATHWAY	0.602256	1.878203	0.027668	0.020043
BIOCARTA_IL12_PATHWAY	0.718206	1.883277	0.035652	0.025827

*TCGA* the cancer genome atlas, *GSEA* gene set enrichment analysis, *COADREAD* colorectal carcinoma.

*GSEA* gene set enrichment analysis.

**Fig. 6:**
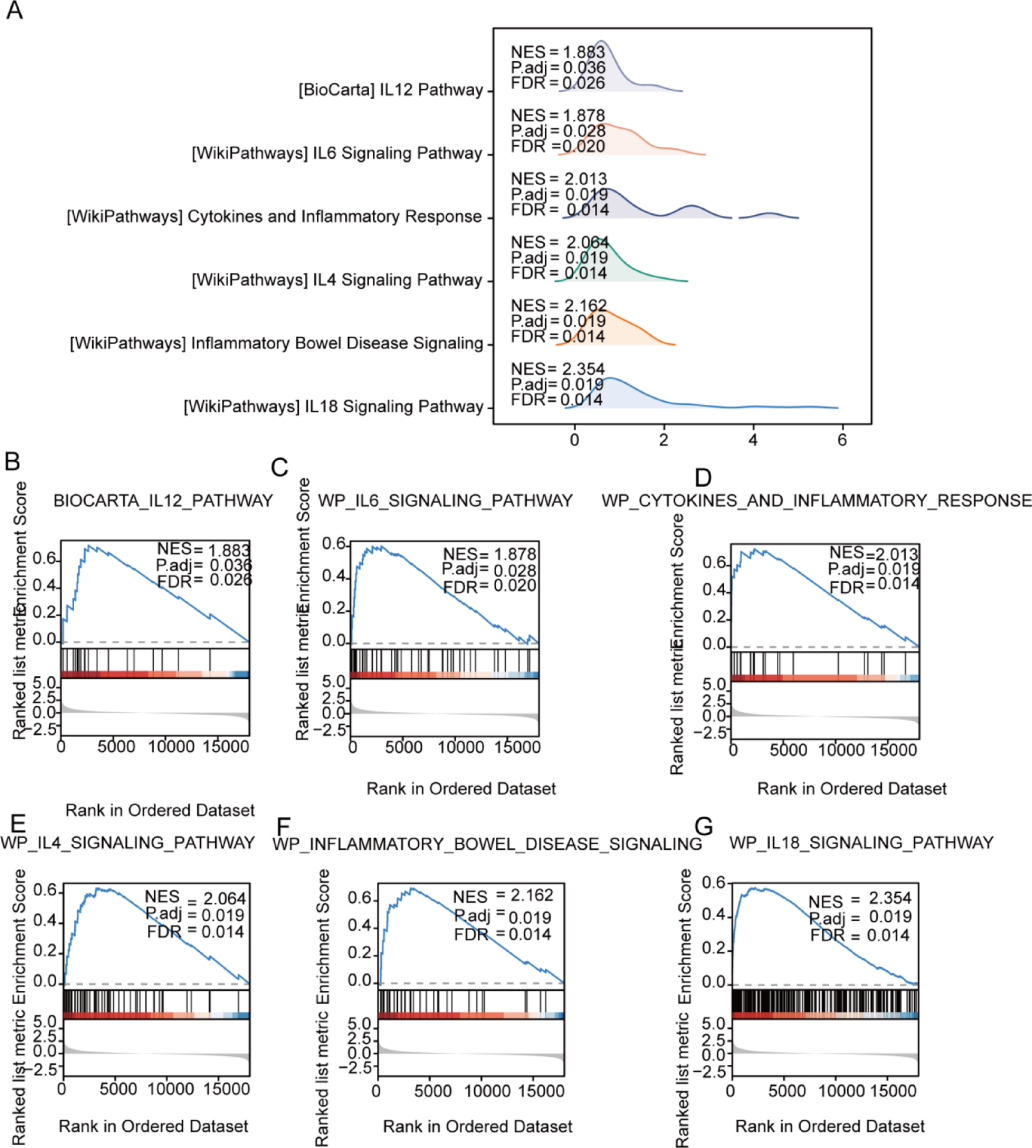
GSEA Enrichment Analysis of GSE4183 Dataset. (**A**) Highlights the primary six biological features of gene enrichment between the disease and control groups (UC/Control) in the GSE4183 dataset. (**B**–**G**) Emphasize the significant enrichment of genes in the GSE4183 dataset within WP_IL18_SIGNALING_PATHWAY (**B**), WP_INFLAMMATORY_BOWEL_DISEASE_SIGNALING (**C**), WP_IL4_SIGNALING_PATHWAY (**D**), WP_CYTOKINES_AND_INFLAMMATORY_RESPONSE (**E**), WP_IL6_SIGNALING_PATHWAY (**F**), and BIOCARTA_IL12_PATHWAY (**G**). GSEA, Gene Set Enrichment Analysis; UC, Ulcerative Colitis. The significant enrichment criteria for GSEA are P.adj < 0.05 and FDR value (q.value) < 0.05.

To assess the impact of gene expression levels between the disease and control groups in the GSE4183 dataset (UC/Control) on UC, we conducted GSEA enrichment analysis. This aimed to investigate the relationship between the expression of all genes within the two groups and their involvement in biological processes, affected cellular components, and molecular functions. The significant enrichment criteria were set at P.adj < 0.05 and FDR value (q.value) < 0.05. The results revealed significant enrichment of genes in the WP_IL18_SIGNALING_PATHWAY (Fig. 6B), WP_INFLAMMATORY_BOWEL_DISEASE_SIGNALING (Fig. 6C), WP_IL4_SIGNALING_PATHWAY (Fig. 6D), WP_CYTOKINES_AND_INFLAMMATORY_RESPONSE (Fig. 6E), WP_IL6_SIGNALING_PATHWAY (Fig. 6F), and BIOCARTA_IL12_PATHWAY (Fig. 6G) pathways (Fig. 6B–G, see Table 4). The GSEA (Gene Set Enrichment Analysis) results are presented as a landscape plot (Fig. 6A).

### Boxplot expression analysis and ROC curves of OXSRDEGs in three datasets

**Fig. 7:**
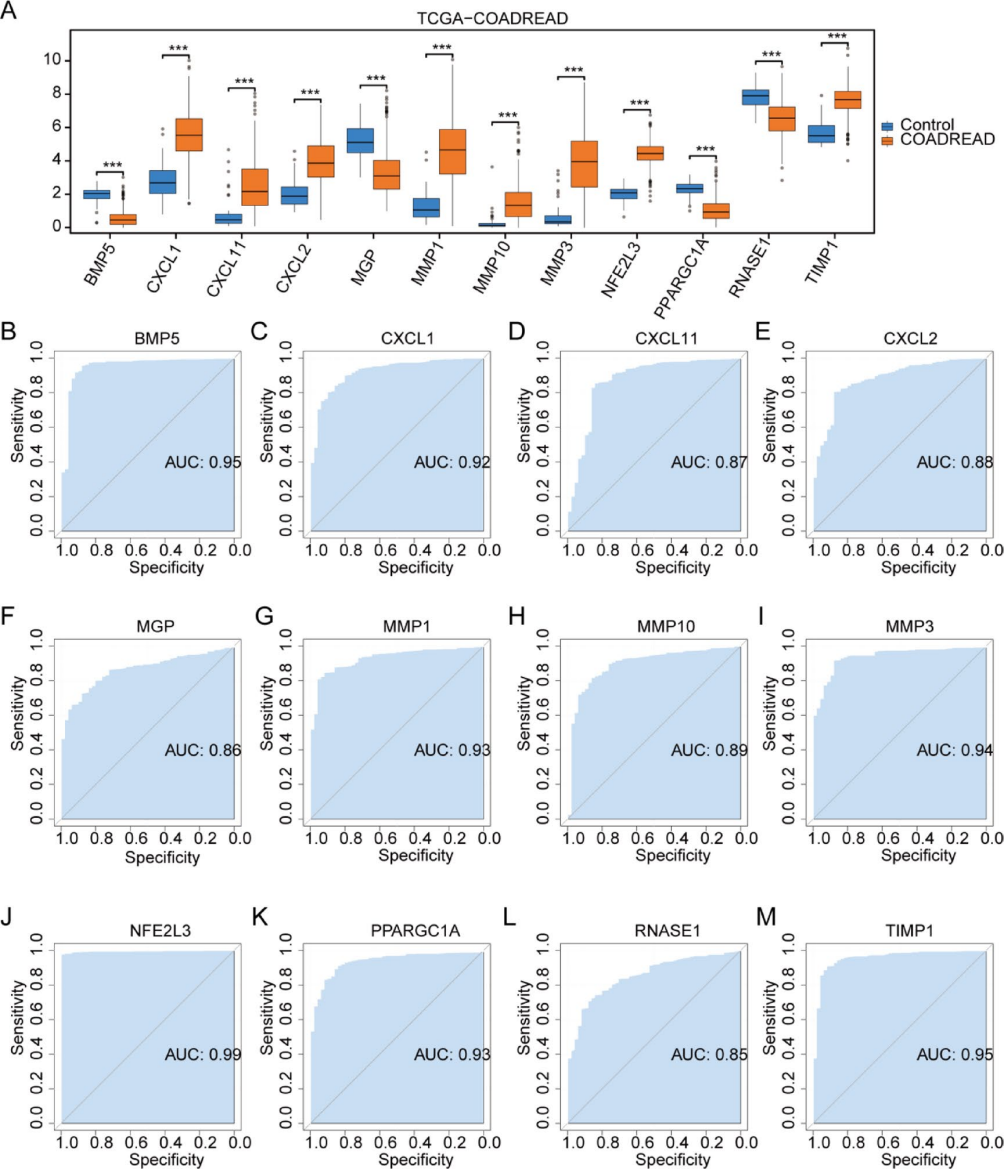
Boxplot Expression Analysis and ROC Curves of OXSRDEGs in TCGA-COADREAD. (**A**) Presents a boxplot illustrating the expression comparison of OXSRDEGs in the TCGA-COADREAD dataset. (**B**–**M**). Showcase ROC curves for individual OXSRDEGs (*BMP5* (**B**), *CXCL1* (**C**), *CXCL11* (**D**), *CXCL2* (**E**), *MGP* (**F**), *MMP1* (**G**), *MMP10* (**H**), *MMP3* (**I**), *NFE2L3* (**J**), *PPARGC1A* (**K**), *RNASE1* (**L**), *TIMP1* (M)) in the TCGA-COADREAD dataset. In the legend, “ns” indicates *P* ≥ 0.05, signifying no statistical significance; *, denoting *P* < 0.05, implies statistical significance; ** indicates *P* < 0.01, representing high statistical significance; *** signifies *P* < 0.001, indicating extremely high statistical significance. A higher AUC in the ROC curve suggests a more effective diagnosis, with AUC between 0.5 and 0.7 indicating low accuracy, AUC between 0.7 and 0.9 indicating moderate accuracy, and AUC above 0.9 indicating high accuracy. TCGA, The Cancer Genome Atlas; COADREAD, Colorectal Carcinoma; OXSRDEGs, Oxidative Stress-Related Differentially Expressed Genes.

The expression analysis of the 12 OXSRDEGs in the TCGA-COADREAD dataset between cancer and control groups revealed significant differences (Fig. 7A). Subsequently, ROC curves were constructed for the 12 OXSRDEGs (COADREAD/Control) to assess their diagnostic potential for CRC. Notably, *BMP5* (Fig. 7B, AUC = 0.956), *CXCL1* (Fig. 7C, AUC = 0.925), *MMP1* (Fig. 7G, AUC = 0.933), *MMP3* (Fig. 7I, AUC = 0.943), *NFE2L3* (Fig. 7J, AUC = 0.996), *PPARGC1A* (Fig. 7K, AUC = 0.939), and *TIMP1* (Fig. 7M, AUC = 0.959) exhibited high accuracy in diagnosing COADREAD. Additionally, *CXCL11* (Fig. 7D, AUC = 0.877), *CXCL2* (Fig. 7E, AUC = 0.883), *MGP* (Fig. 7F, AUC = 0.868), *MMP10* (Fig. 7H, AUC = 0.899), and *RNASE1* (Fig. 7L, AUC = 0.857) demonstrated moderate accuracy in diagnosing CRC.

**Fig. 8:**
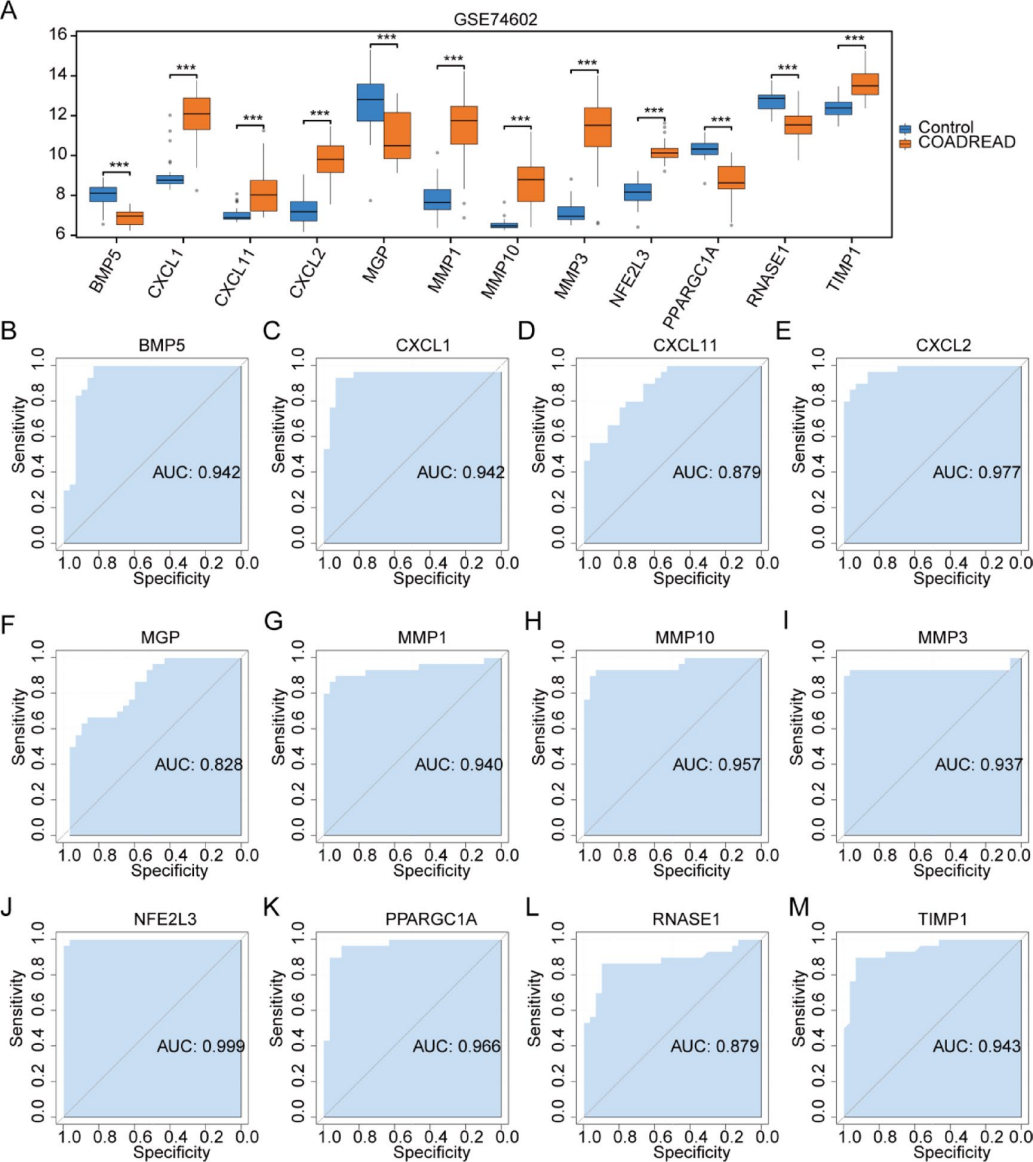
Boxplot Expression Analysis and ROC Curves of OXSRDEGs in GSE74602. (**A**) The boxplot comparison of OXSRDEGs’ expression in the GSE74602 dataset (COADREAD/Control) is illustrated. B-M. The ROC curves for OXSRDEGs (*BMP5* (**B**), *CXCL1* (**C**), *CXCL11* (**D**), *CXCL2* (**E**), *MGP* (**F**), *MMP1* (**G**), *MMP10* (**H**), *MMP3*(I), *NFE2L3*(**J**), *PPARGC1A* (**K**), *RNASE1*(**L**), *TIMP1*(**M**)) in the GSE4183 dataset are presented. ‘ns’ indicates *P* ≥ 0.05, denoting no statistical significance; * denotes *P* < 0.05, indicating statistical significance; ** signifies *P* < 0.01, representing high statistical significance; *** indicates *P* < 0.001, highlighting extremely high statistical significance. The proximity of the AUC in the ROC curve to 1 correlates with improved diagnostic performance. An AUC ranging from 0.5 to 0.7 suggests low accuracy; an AUC between 0.7 and 0.9 indicates moderate accuracy; an AUC exceeding 0.9 signifies high accuracy. OXSRDEGs stands for Oxidative Stress Related Differentially Expressed Genes; COADREAD denotes Colorectal Carcinoma; UC refers to Ulcerative Colitis.

The expression analysis of the 12 OXSRDEGs in the GSE74602 dataset between different groups also demonstrated significant differences (Fig. 8A). ROC curves were generated for the 12 OXSRDEGs (COADREAD/Control) in the GSE74602 dataset to assess their diagnostic potential for CRC. Among them, *BMP5* (Fig. 8B, AUC = 0.942), *CXCL1* (Fig. 8C, AUC = 0.942), *CXCL2* (Fig. 8E, AUC = 0.977), *MMP1* (Fig. 8G, AUC = 0.940), *MMP10* (Fig. 8H, AUC = 0.957), *MMP3* (Fig. 8I, AUC = 0.937), *NFE2L3* (Fig. 8J, AUC = 0.999), *PPARGC1A* (Fig. 8K, AUC = 0.966), and *TIMP1* (Fig. 8M, AUC = 0.943) exhibited high accuracy in diagnosing COADREAD. Additionally, *CXCL11* (Fig. 8D, AUC = 0.879), *MGP* (Fig. 8F, AUC = 0.828), and *RNASE1* (Fig. 8L, AUC = 0.879) demonstrated moderate accuracy in diagnosing CRC.

**Fig. 9:**
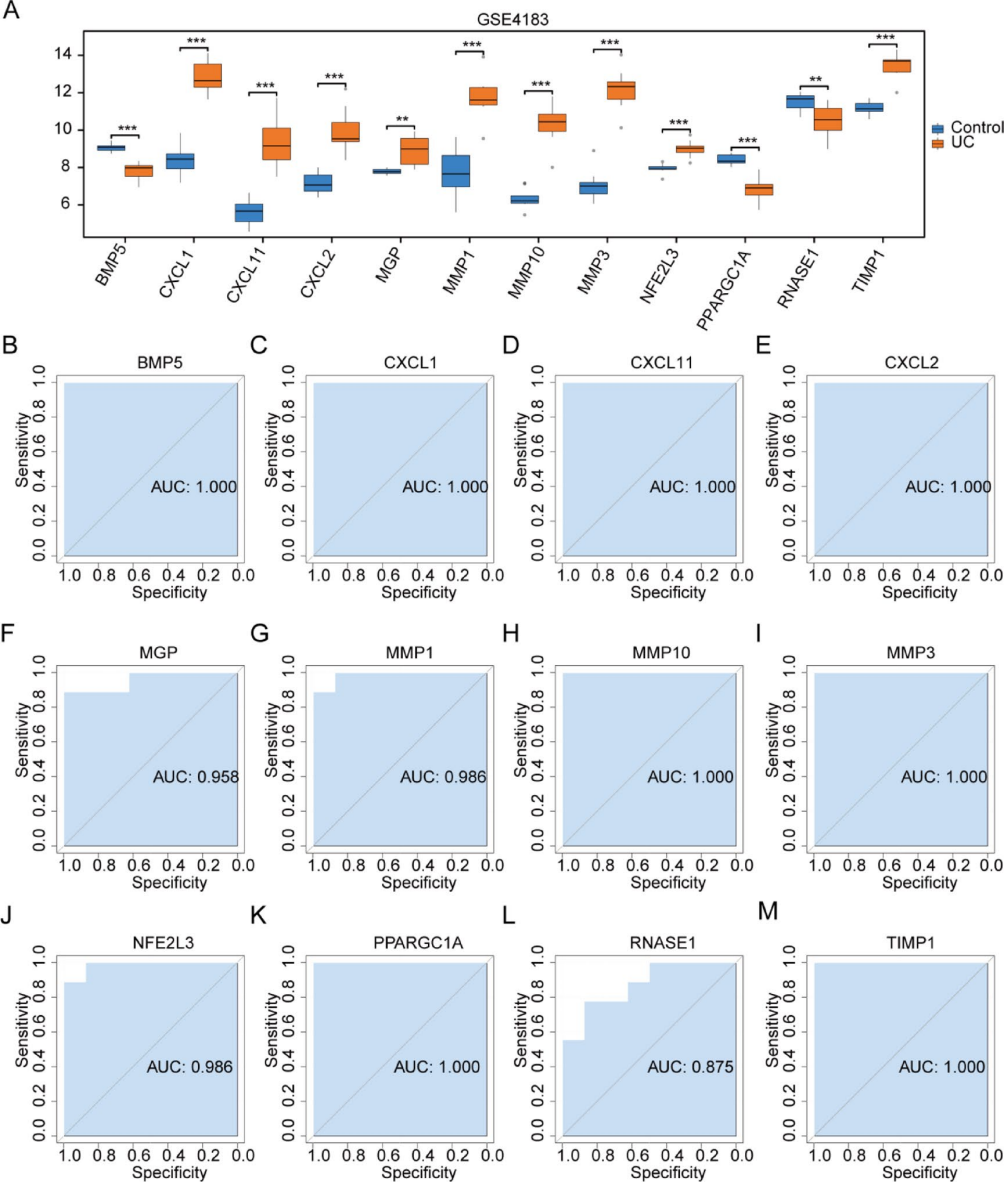
Boxplot Expression Analysis and ROC Curves of OXSRDEGs in GSE4183. (**A**) The boxplot comparison of OXSRDEGs’ expression in the GSE4183 dataset (COADREAD/Control) is illustrated. (**B**–**M**) The ROC curves for OXSRDEGs (*BMP5*(**B**), *CXCL1*(**C**), *CXCL11*(**D**), *CXCL2*(**E**), *MGP* (**F**), *MMP1*(**G**), *MMP10*(**H**), *MMP3*(I), *NFE2L3*(**J**), *PPARGC1A* (**K**), *RNASE1*(**L**), *TIMP1*(**M**)) in the GSE4183 dataset are presented. ‘ns’ indicates *P* ≥ 0.05, denoting no statistical significance; * denotes *P* < 0.05, indicating statistical significance; ** signifies *P* < 0.01, representing high statistical significance; *** indicates *P* < 0.001, highlighting extremely high statistical significance. The proximity of the AUC in the ROC curve to 1 correlates with improved diagnostic performance. An AUC ranging from 0.5 to 0.7 suggests low accuracy; an AUC between 0.7 and 0.9 indicates moderate accuracy; an AUC exceeding 0.9 signifies high accuracy. OXSRDEGs stands for Oxidative Stress Related Differentially Expressed Genes; COADREAD denotes Colorectal Carcinoma; UC refers to Ulcerative Colitis.

The expression analysis of the 12 OXSRDEGs in the GSE4183 dataset between different groups also demonstrated significant differences (Fig. 9A). ROC curves were generated for the 12 OXSRDEGs (UC/Control) in the GSE4183 dataset to assess their diagnostic potential for UC. Among them, *BMP5* (Fig. 9B, AUC = 1.000), *CXCL1* (Fig. 9C, AUC = 1.000), *CXCL11* (Fig. 9D, AUC = 1.000),*CXCL3* (Fig. 9E, AUC = 1.000), *MGP* (Fig. 9F, AUC = 0.958), *MMP1* (Fig. 9G, AUC = 0.986), *MMP10* (Fig. 9H, AUC = 1.000), *MMP3* (Fig. 9I, AUC = 1.000), *NFE2L3* (Fig. 9J, AUC = 0.986), *PPARGC1A* (Fig. 9K, AUC = 1.000), and *TIMP1* (Fig. 9M, AUC = 1.000) exhibited high accuracy in diagnosing UC. Additionally, *RNASE1* (Fig. 9L, AUC = 0.875) demonstrated moderate accuracy in diagnosing UC.

**Fig. 10:**
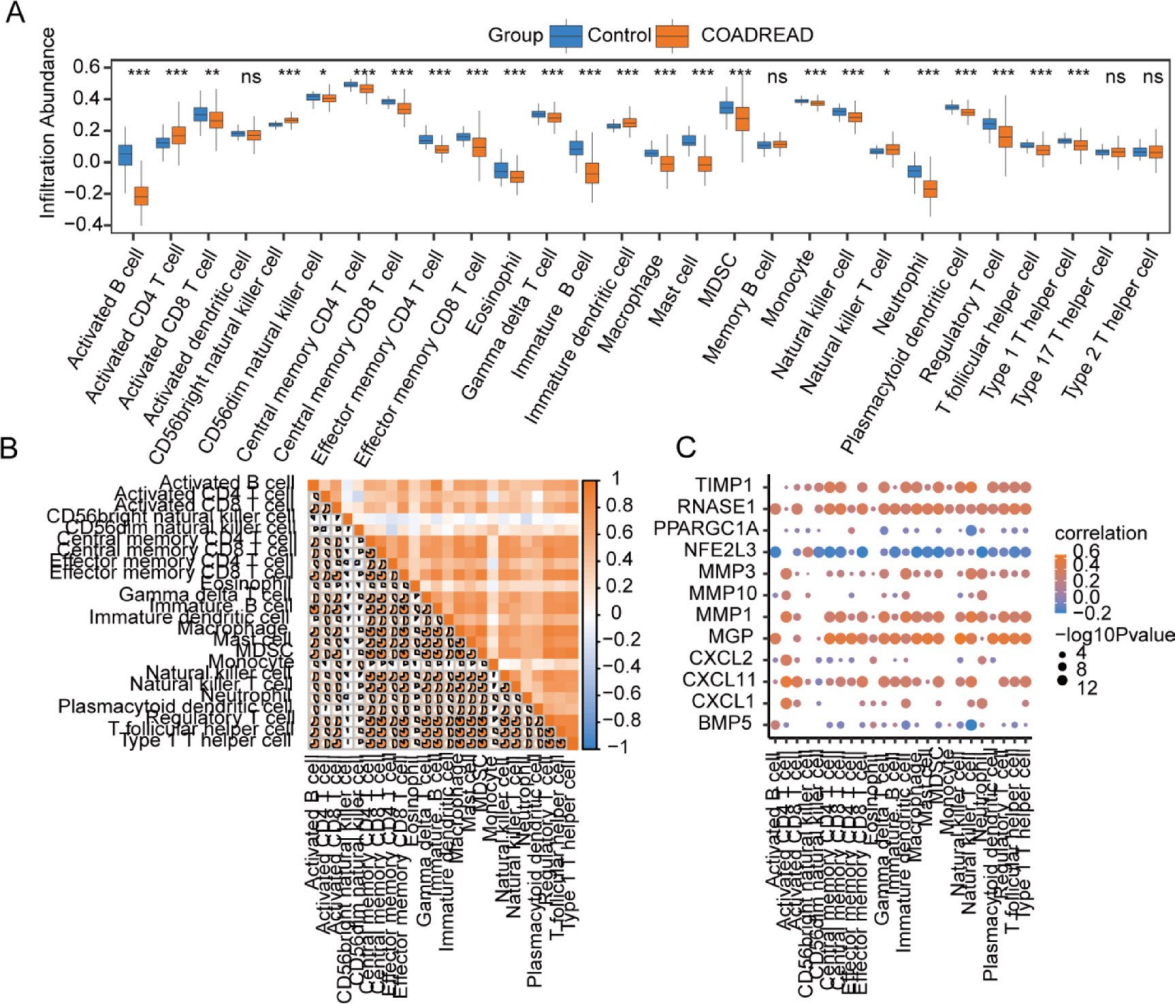
SSGSEA Immune Characteristics Differences Analysis in TCGA-COADREAD Dataset. (**A**) The SSGSEA immune infiltration analysis comparison diagram between groups in the TCGA-COADREAD dataset. (**B**) The correlation analysis of immune cells showing significant differences between groups in the TCGA-COADREAD dataset. (**C**) The correlation dot plot highlighting the relationship between immune cells with significant differences and OXSRDEGs. The notation ‘ns’ corresponds to *P* ≥ 0.05, indicating no statistical significance; * denotes *P* < 0.05, signifying statistical significance; ** represents *P* < 0.01, marking high statistical significance; *** stands for *P* < 0.001, showing extremely high statistical significance. TCGA refers to The Cancer Genome Atlas; COADREAD to Colorectal Carcinoma; SSGSEA to Single-Sample Gene-Set Enrichment Analysis; OXSRDEGs to Oxidative Stress Related Differentially Expressed Genes.

To investigate the immune infiltration disparities between the two groups within the TCGA-COADREAD dataset, the SSGSEA algorithm was employed to assess the infiltration extent of 28 immune cell types in both sample sets. Subsequently, the Mann–Whitney U test analyzed the infiltration discrepancies between the groups, culminating in a comparative visualization (Fig. 10A). The analysis revealed that 24 immune cell types exhibited statistically significant differences (*P* < 0.05) between the two groups, namely Activated B cell, Activated CD4 T cell, Activated CD8 T cell, CD56bright natural killer cell, CD56dim natural killer cell, Central memory CD4 T cell, Central memory CD8 T cell, Effector memory CD4 T cell, Effector memory CD8 T cell, Eosinophil, Gamma delta T cell, Immature B cell, Immature dendritic cell, Macrophage, Mast cell, MDSC, Monocyte, Natural killer cell, Natural killer T cell, Neutrophil, Plasmacytoid dendritic cell, Regulatory T cell, T follicular helper cell, Type 1 T helper cell.

The Spearman statistical algorithm was applied to further verify the correlation between the infiltration abundance of these 24 significantly different immune cells within the TCGA-COADREAD dataset (Fig. 10B). The findings indicated a positive correlation among most of the 24 immune cells, with the correlation between MDSC and Macrophage being especially notabe (Fig. 10B).

Additionally, the Spearman statistical algorithm evaluated the correlation between the infiltration abundance of these 24 immune cells and the expression levels of the 12 OXSRDEGs, using a screening threshold of *P* < 0.05 to select significant correlations for representation in a correlation dot plot (Fig. 10C). The results demonstrated that significant positive correlations were found for *MGP, MMP1, MMP3, RNASE1*, and *TIMP1* with the immune cells, with the strongest correlation observed between Natural killer cells and *MGP*.

**Fig. 11:**
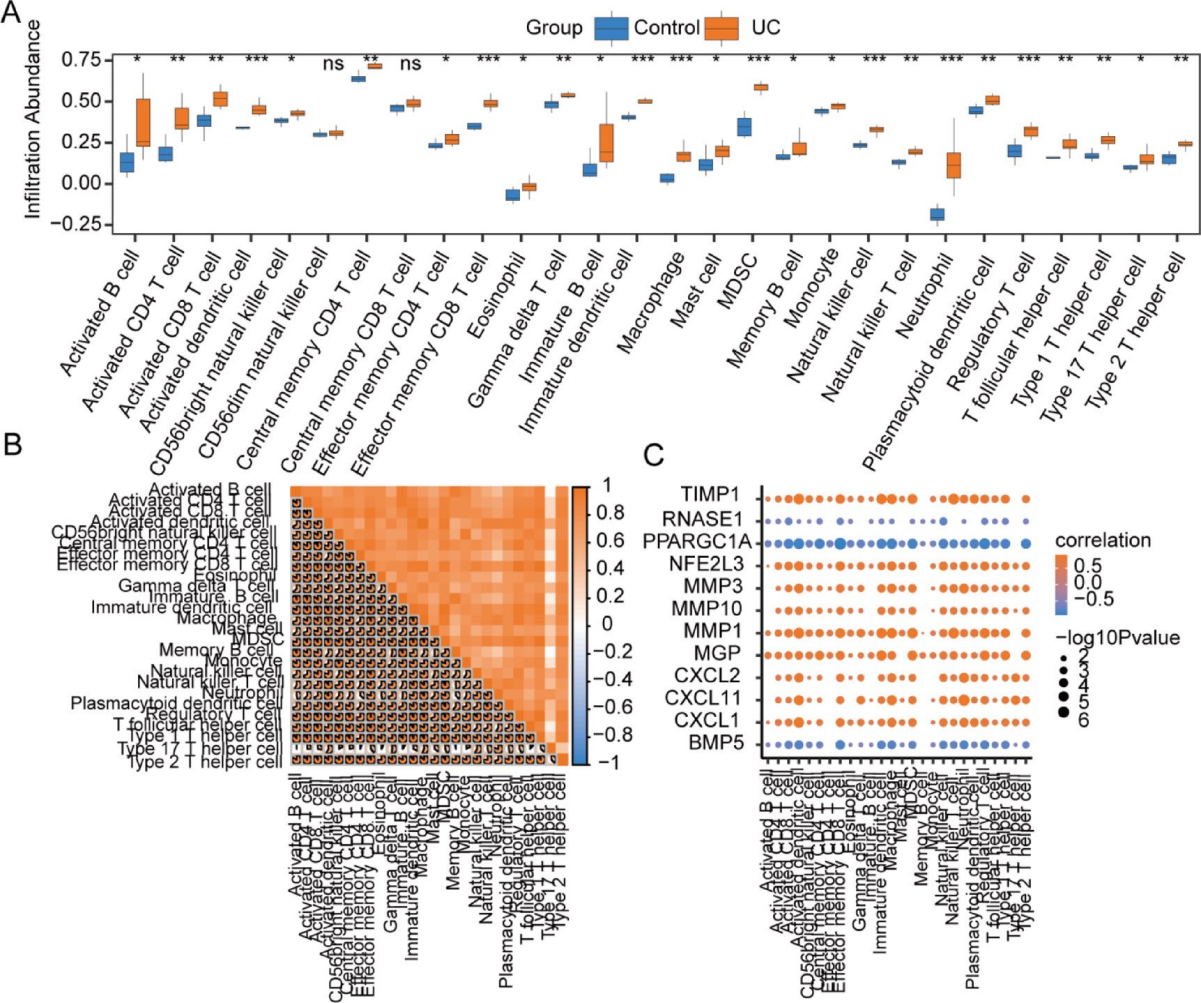
SSGSEA Immune Characteristics in GSE4183 Dataset. (**A**) The SSGSEA immune infiltration analysis comparison displays the differences in immune cell infiltration between groups within the GSE4183 dataset. (**B**) The analysis of correlations among immune cells exhibiting significant differences between groups in the GSE4183 dataset. (**C**) The correlation dot plot illustrates the relationships between significantly differing immune cells and OXSRDEGs in the GSE4183 dataset. ‘ns’ denotes *P* ≥ 0.05, indicating a lack of statistical significance; * represents *P* < 0.05, signifying statistical significance; ** signifies *P* < 0.01, indicating high statistical significance; *** stands for *P* < 0.001, denoting extremely high statistical significance. SSGSEA stands for Single-Sample Gene-Set Enrichment Analysis; OXSRDEGs refers to Oxidative Stress Related Differentially Expressed Genes; UC stands for Ulcerative Colitis.

Within the GSE4183 dataset, the SSGSEA algorithm calculated the infiltration abundance of 28 immune cell types in two sample groups. Subsequently, the Mann–Whitney U test analyzed the differences in infiltration between these groups, resulting in a comparative diagram (Fig. 11A). The findings indicated that 26 immune cell types exhibited statistically significant differences (*P* < 0.05) between the groups, including Activated B cell, Activated CD4 T cell, Activated CD8 T cell, Activated dendritic cell, CD56bright natural killer cell, Central memory CD4 T cell, Effector memory CD4 T cell, Effector memory CD8 T cell, Eosinophil, Gamma delta T cell, Immature B cell, Immature dendritic cell, Macrophage, Mast cell, MDSC, Memory B cell, Monocyte, Natural killer cell, Natural killer T cell, Neutrophil, Plasmacytoid dendritic cell, Regulatory T cell, T follicular helper cell, Type 1 T helper cell, Type 17 T helper cell, Type 2 T helper cell.

The Spearman statistical algorithm further assessed the correlation between the infiltration abundance of these 26 distinct immune cells in the GSE4183 dataset (Fig. 11B). Except for a negative correlation between Type 17 T helper cell and Memory B cell, all other cells demonstrated positive correlations, with the strongest correlation observed between Regulatory T cell and Effector memory CD8 T cell (Fig. 11B).

Moreover, the Spearman statistical algorithm evaluated the correlation between the infiltration extent of the aforementioned 26 immune cells and the expression levels of the 12 OXSRDEGs, employing a significance threshold of *P* < 0.05 for selection. The correlation dot plot was then created (Fig. 11C). The analysis revealed that *BMP5*, *PPARGC1A*, and *RNASE1* exhibited significant negative correlations with immune cells, whereas *CXCL1, CXCL11, CXCL2, MGP, MMP1, MMP10, MMP3, NFE2L3*, and *TIMP1* showed significant positive correlations. Notably, the strongest correlation was between Effector memory CD8 T cell and *PPARGC1A*.

### Immune feature difference analysis of TCGA-COADREAD dataset and GSE4183 dataset using CIBERSORT

**Fig. 12:**
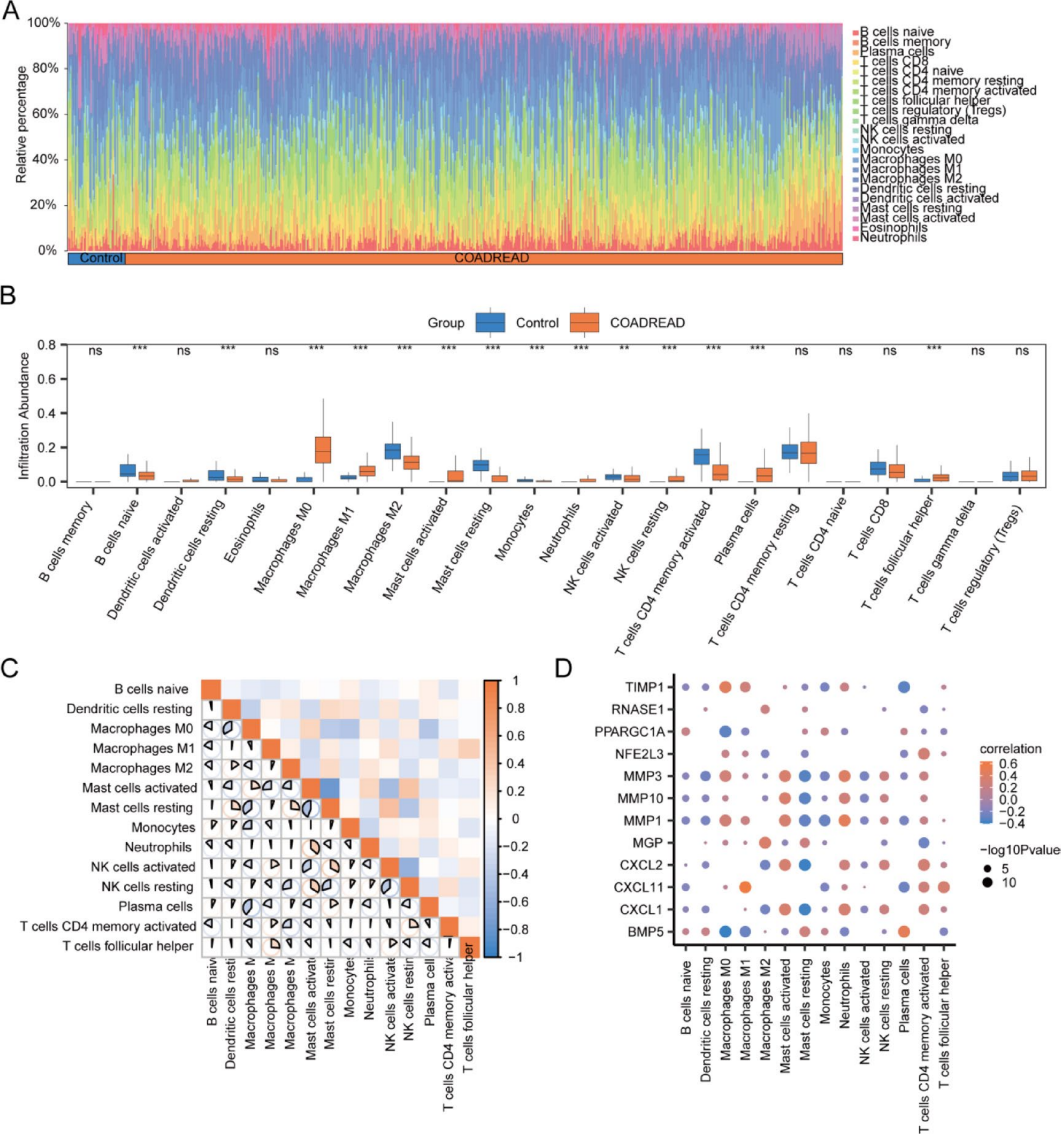
Difference analysis of CIBERSORT immune features between cancer control groups in TCGA-COADREAD dataset. (**A**, **B**) The stacked bar chart illustrates the CIBERSORT immune infiltration analysis outcomes for 22 immune cell types between groups in the TCGA-COADREAD dataset (**A**), alongside a grouped comparison chart (**B**). (**C**) This section presents the correlation analysis of immune cells that exhibit statistically significant differences between groups in the TCGA-COADREAD dataset. (**D**) The correlation dot plot depicts the relationships between immune cells with statistically significant differences between groups in the TCGA-COADREAD dataset and OXSRDEGs. The notation ‘ns’ corresponds to *P* ≥ 0.05, indicating no statistical significance; * denotes *P* < 0.05, signifying statistical significance; ** signifies *P* < 0.01, indicating high statistical significance; *** represents *P* < 0.001, conveying extremely high statistical significance. TCGA stands for The Cancer Genome Atlas; COADREAD represents Colorectal Carcinoma; OXSRDEGs refers to Oxidative Stress Related Differentially Expressed Genes.

The CIBERSORT algorithm was employed to evaluate the infiltration extent of 22 immune cell types across two groups in the TCGA-COADREAD dataset, with their infiltration proportions depicted through a stacked bar chart (Fig. 12A). The analysis revealed that the infiltration levels of the 22 immune cell types in TCGA-COADREAD dataset samples varied, with none showing an infiltration level of zero.

The differences in infiltration level among the 22 immune cell types were further illustrated via a grouped comparison chart. This analysis demonstrated that 14 immune cell types exhibited statistically significant differences in infiltration levels between the groups (*P* < 0.05), including B cells naive, Dendritic cells resting, Macrophages M0, Macrophages M1, Macrophages M2, Mast cells activated, Mast cells resting, Monocytes, Neutrophils, NK cells activated, NK cells resting, Plasma cells, T cells CD4 memory activated, and T cells follicular helper (Fig. 12B).

The Spearman statistical algorithm assessed the correlation in infiltration level among these 14 types of immune cells, revealing an equal distribution of positive and negative correlations (Fig. 12C). Notably, the strongest correlation was observed between activated Mast cells and resting Mast cells. Additionally, the correlation between the infiltration abundance of these 14 immune cells and the 12 OXSRDEGs was analyzed, with significant correlations identified using a threshold of *P* < 0.05 for inclusion in the correlation dot plot (Fig. 12D). Among these, the strongest correlation was found between Macrophages M1 and *CXCL11*.

**Fig. 13:**
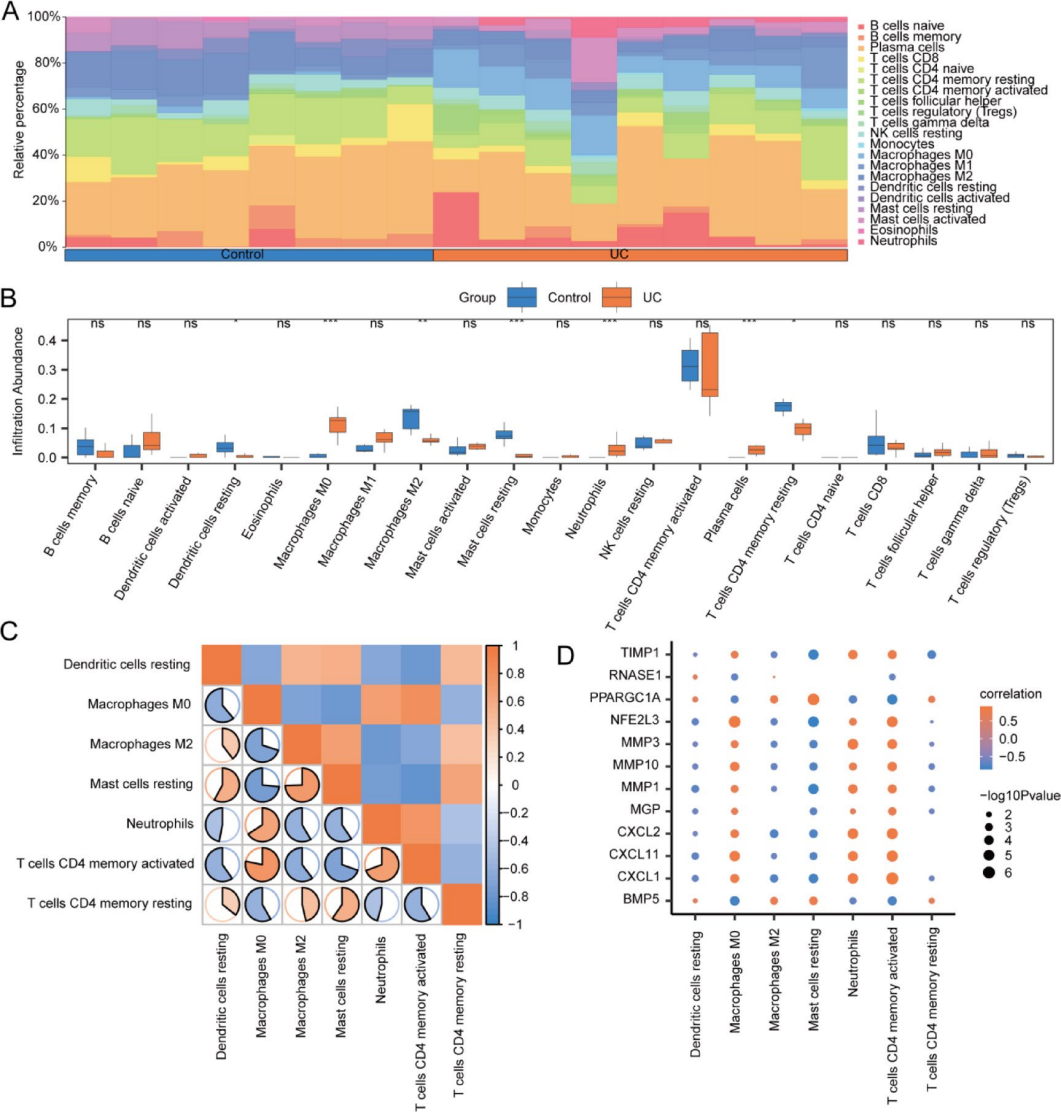
Difference analysis of CIBERSORT immune features between disease control groups in GSE4183 dataset. (**A**, **B**) The stacked bar chart illustrates the results of the CIBERSORT immune infiltration analysis, detailing the distribution of 22 immune cell types between groups in the GSE4183 dataset (**A**), accompanied by a grouped comparison chart (**B**). (**C**) The section provides a correlation analysis of immune cells that exhibited statistically significant differences between groups in the GSE4183 dataset. (**D**) The correlation dot plot showcases the relationships between immune cells with statistically significant differences between groups in the GSE4183 dataset and OXSRDEGs. ‘ns’ indicates *P* ≥ 0.05, representing no statistical significance; * denotes *P* < 0.05, signaling statistical significance; ** signifies *P* < 0.01, indicating high statistical significance; *** stands for *P* < 0.001, denoting extremely high statistical significance. OXSRDEGs are defined as Oxidative Stress Related Differentially Expressed Genes; UC refers to Ulcerative Colitis.

Utilizing the CIBERSORT algorithm, the study quantified the infiltration level of 22 immune cell types between groups in the GSE4183 dataset, displaying the proportional abundance of immune cells in samples via a stacked bar chart (Fig. 13A). The analysis revealed that 21 immune cell types in the GSE4183 dataset samples exhibited non-zero infiltration levels, including B cells memory, B cells naïve, Dendritic cells activated, Dendritic cells resting, Eosinophils, Macrophages M0, Macrophages M1, Macrophages M2, Mast cells activated, Mast cells resting, Monocytes, Neutrophils, NK cells resting, Plasma cells, T cells CD4 memory activated, T cells CD4 memory resting, T cells CD4 naïve, T cells CD8, T cells follicular helper, T cells gamma delta, and regulatory T cells (Tregs).

The study identified statistically significant differences (*P* < 0.05) in the infiltration level of 7 immune cell types between groups in the GSE4183 dataset: Dendritic cells resting, Macrophages M0, Macrophages M2, Mast cells resting, Neutrophils, T cells CD4 memory activated, and T cells CD4 memory resting (Fig. 13B).

The Spearman statistical algorithm was applied to analyze the correlation in infiltration level among these 7 immune cell types with significant differences between groups in the GSE4183 dataset (Fig. 13C). The findings indicated an equal distribution of positive and negative correlations among these 7 immune cells, with the strongest correlation observed between Macrophages M0 and T cells CD4 memory activated.

Furthermore, the Spearman statistical algorithm assessed the correlation in infiltration abundance of these 7 immune cells with significant differences between groups in the GSE4183 dataset and the 12 OXSRDEGs (Fig. 13D). The analysis demonstrated a balanced number of positive and negative correlations, with the most significant correlation identified between T cells CD4 memory activated and *CXCL1*.

### Construction of a multivariate cox prognostic model for the TCGA-COADREAD dataset

**Fig. 14:**
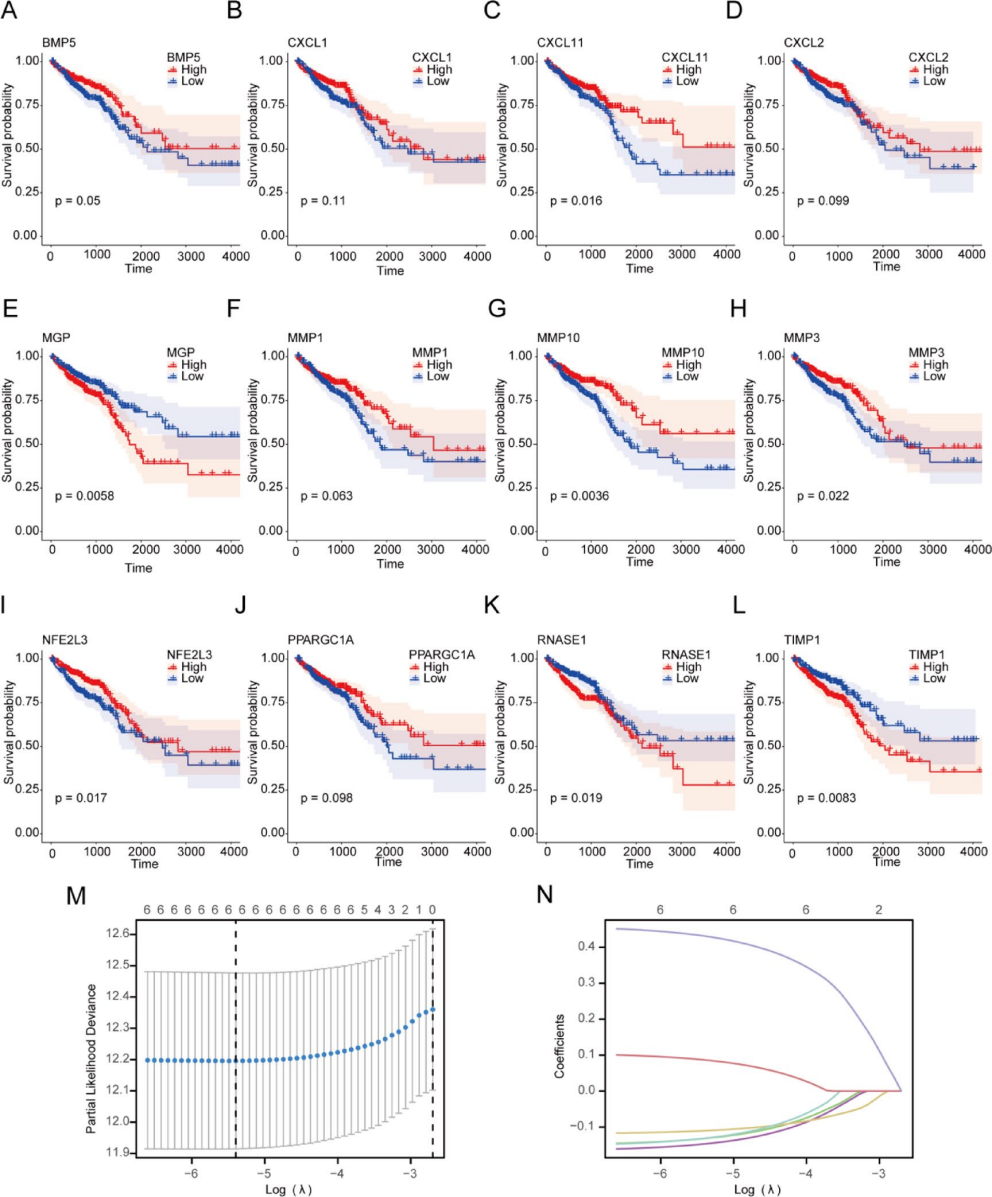
Survival Kaplan–Meier Curve Ff OXSRDEGs. (**A**–**L**) The study presents an analysis of 12 Oxidative Stress Related Differentially Expressed Genes (OXSRDEGs), including *BMP5* (**A**), *CXCL*1 (**B**), *CXCL1*1 (**C**), *CXCL2* (**D**), *MGP* (**E**), *MMP1* (**F**), *MMP10* (**G**), *MMP3* (**H**), *NFE2L3* (**I**), *PPARGC1A* (**J**), *RNASE1* (**K**), and *TIMP1* (**L**)). (**M**, **N**) It showcases a prognostic model based on the results of LASSO regression analysis of OXSRDEGs (**M**) and a variable trajectory diagram (**N**). OXSRDEGs stand for Oxidative Stress Related Differentially Expressed Genes; KM represents Kaplan–Meier survival analysis; LASSO denotes the Least Absolute Shrinkage and Selection Operator technique.

Prior to the development of the Cox prognostic model for the TCGA-COADREAD dataset, the study combined data on overall survival (OS), OS time, and the expression levels of 12 OXSRDEGs (*BMP5, CXCL1, CXCL11, CXCL2, MGP, MMP1, MMP10, MMP3, NFE2L3, PPARGC1A, RNASE1, TIMP1*) from samples in the TCGA-COADREAD dataset to generate survival curves (Kaplan–Meier curves) (Fig. 14A–L). Of these, *CXCL11* (Fig. 14C, p = 0.016), *MGP* (Fig. 14E, p = 0.0058), *MMP10* (Fig. 14G, p = 0.0036), *MMP3* (Fig. 14H, p = 0.022), *NFE2L3* (Fig. 14I, p = 0.017), *RNASE1* (Fig. 14K, p = 0.019), and *TIMP1* (Fig. 14L, p = 0.0083) demonstrated significant differences in overall survival (OS) between groups with high and low expressions (divided at the median expression level).

To assess the prognostic value of the 7 OXSRDEGs within the TCGA-COADREAD dataset, LASSO regression analysis was performed on these genes. This analysis resulted in the retention of 6 out of the 7 OXSRDEGs: *CXCL11, MMP10, MMP3, NFE2L3, RNASE1*, and *TIMP1* (Full names of genes are shown in Table 5). These genes were then utilized as Prognostic OXSRDEGs for the subsequent construction of the multivariate Cox model (Fig. 14M,N).

**Table 5:** Table of hub genes.

Hub genes	Full name
CXCL11	C-X-C Motif Chemokine Ligand 11
MMP10	Matrix Metallopeptidase 10
MMP3	Matrix Metallopeptidase 3
NFE2L3	NFE2 Like BZIP Transcription Factor 3
RNASE1	Ribonuclease A Family Member 1, Pancreatic
TIMP1	TIMP Metallopeptidase Inhibitor 1

**Fig. 15:**
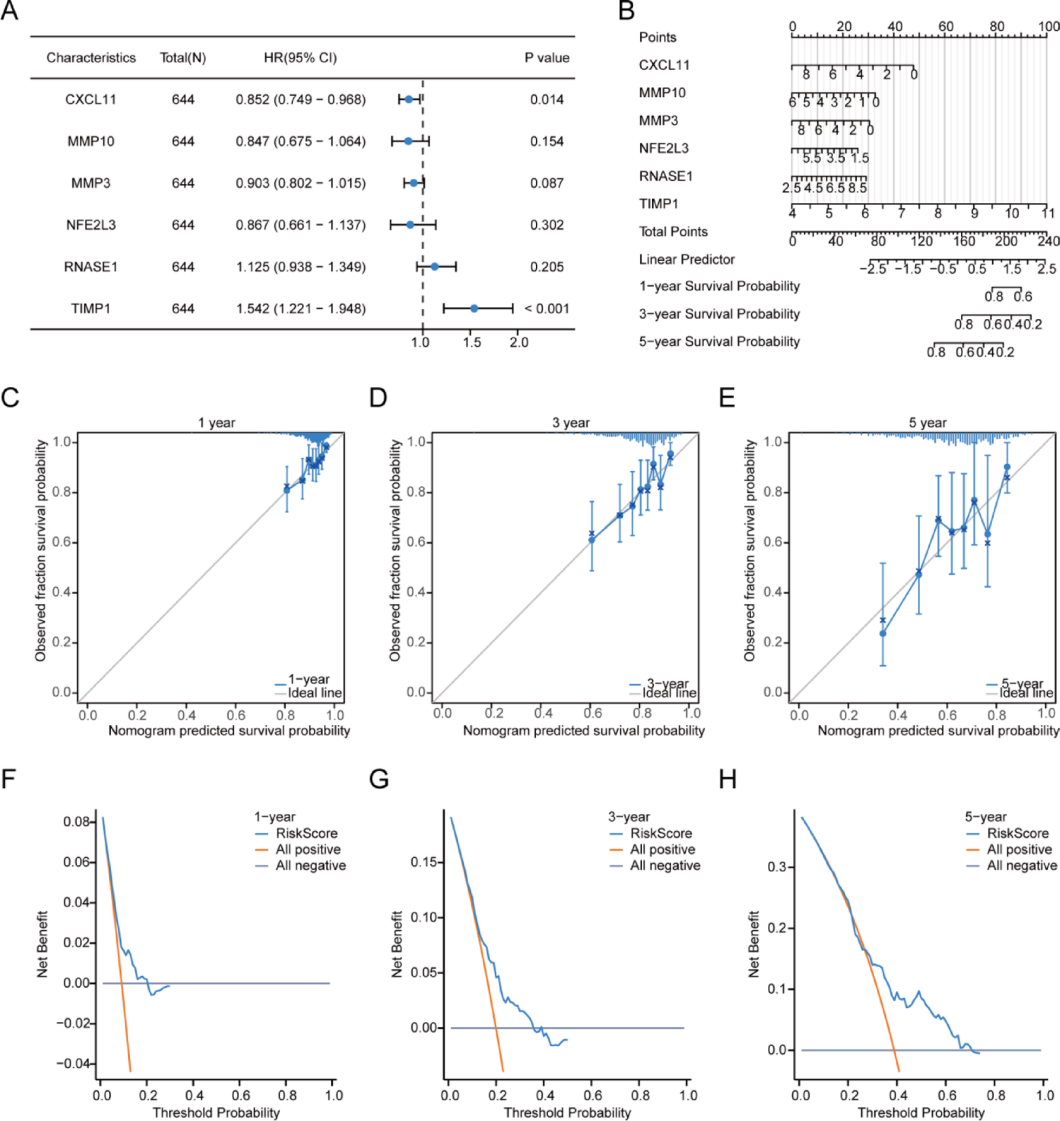
Construction of multivariate Cox regression model in TCGA-COADREAD dataset. (**A**) The study presents a forest plot of the multivariate Cox regression model for the TCGA-COADREAD dataset, offering a comprehensive visualization of the prognostic significance of various factors. (**B**) It also introduces a nomogram based on the multivariate Cox regression model, designed to predict survival rates. (**C**–**E**) The calibration curves for the nomogram, assessing its accuracy in predicting 1-year (**C**), 3-year (**D**), and 5-year (**E**) survival rates, are meticulously plotted. (**F**–**H**) Decision curve analysis (DCA) is employed to evaluate the clinical utility of the model for predicting 1-year (**F**), 3-year (**G**), and 5-year (**H**) survival rates, with statistical significance thresholds indicated for P values: *P* ≥ 0.05 implies no statistical significance; *P* < 0.05 indicates statistical significance; *P* < 0.01 denotes high statistical significance; *P* < 0.001 signifies extremely high statistical significance. TCGA stands for The Cancer Genome Atlas; COADREAD refers to Colorectal Carcinoma; OXSRDEGs are Oxidative Stress Related Differentially Expressed Genes; DCA represents Decision Curve Analysis.

Subsequently, the clinical data of COADREAD samples from the TCGA-COADREAD dataset were statistically evaluated (with detailed patient baseline information displayed in Table 6). This analysis incorporated the 6 prognostic OXSRDEGs (*CXCL11, MMP10, MMP3, NFE2L3, RNASE1, TIMP1*) into a multivariate Cox regression analysis to develop a comprehensive Cox prognostic model. The findings from the multivariate Cox regression analysis were then compiled and presented in a forest plot (Fig. 15A).

$$\begin{aligned} {\text{RiskScore}} & { } = - 2.395546422 + CXCL11{*} - 0.160680968 + MMP10{*} \\ & \quad - 0.165561724 + MMP3{*} - 0.102576422{ } + NFE2L3{*} \\ & \quad - 0.142959625 + RNASE1{*}0.117571474 + TIMP1{*}0.433351165{ } \\ \end{aligned}$$

**Table 6:** Patient characteristics of TCGA-COADREAD.

Characteristics	Overall
Pathologic T stage, n (%)	
T1&T2	131 (20.4%)
T3	436 (68%)
T4	74 (11.5%)
Pathologic N stage, n (%)	
N0	368 (57.5%)
N1	153 (23.9%)
N2	119 (18.6%)
Pathologic M stage, n (%)	
M0	475 (84.2%)
M1	89 (15.8%)
OS event, n (%)	
Alive	515 (80%)
Dead	129 (20%)

*TCGA* the cancer genome atlas, *COADREAD* colorectal carcinoma.

The predictive performance of the model was further assessed using nomogram analysis, leading to the creation of a nomogram (Fig. 15B). A nomogram, or a graphical representation, translates multivariate regression analysis outcomes into a scoring system that quantifies each variable’s impact within the model, facilitating the prediction of event occurrence probabilities.

Moreover, prognostic calibration analysis for 1-year (Fig. 15C), 3-year (Fig. 15D), and 5-year (Fig. 15E) outcomes was conducted for the nomogram of the multivariate Cox prognostic model, resulting in calibration curve graphs (Fig. 15C–E). In these graphs, the x-axis indicates the model’s predicted survival probability, while the y-axis shows the actual survival probability derived from the data. Different colored lines and markers symbolize predictions at varying time points, with proximity to the gray ideal line indicating improved prediction accuracy.

Finally, decision curve analysis (DCA) was utilized to examine the clinical utility of the constructed multivariate Cox regression model over 1 year (Fig. 15F), 3 years (Fig. 15G), and 5 years (Fig. 15H) (Fig. 15F–H). The DCA graph’s x-axis represents the probability threshold, and the y-axis measures the net benefit. The effectiveness of the model is determined by the area between the model’s curve and the baseline, with a larger area indicating superior model performance. The analysis suggests that the clinical predictive capacity of the constructed multivariate Cox regression model improves over time, ranking as 5 years > 3 years > 1 year.

### Construction of PPI network and mRNA-TF, mRNA-miRNA interaction networks

**Fig. 16:**
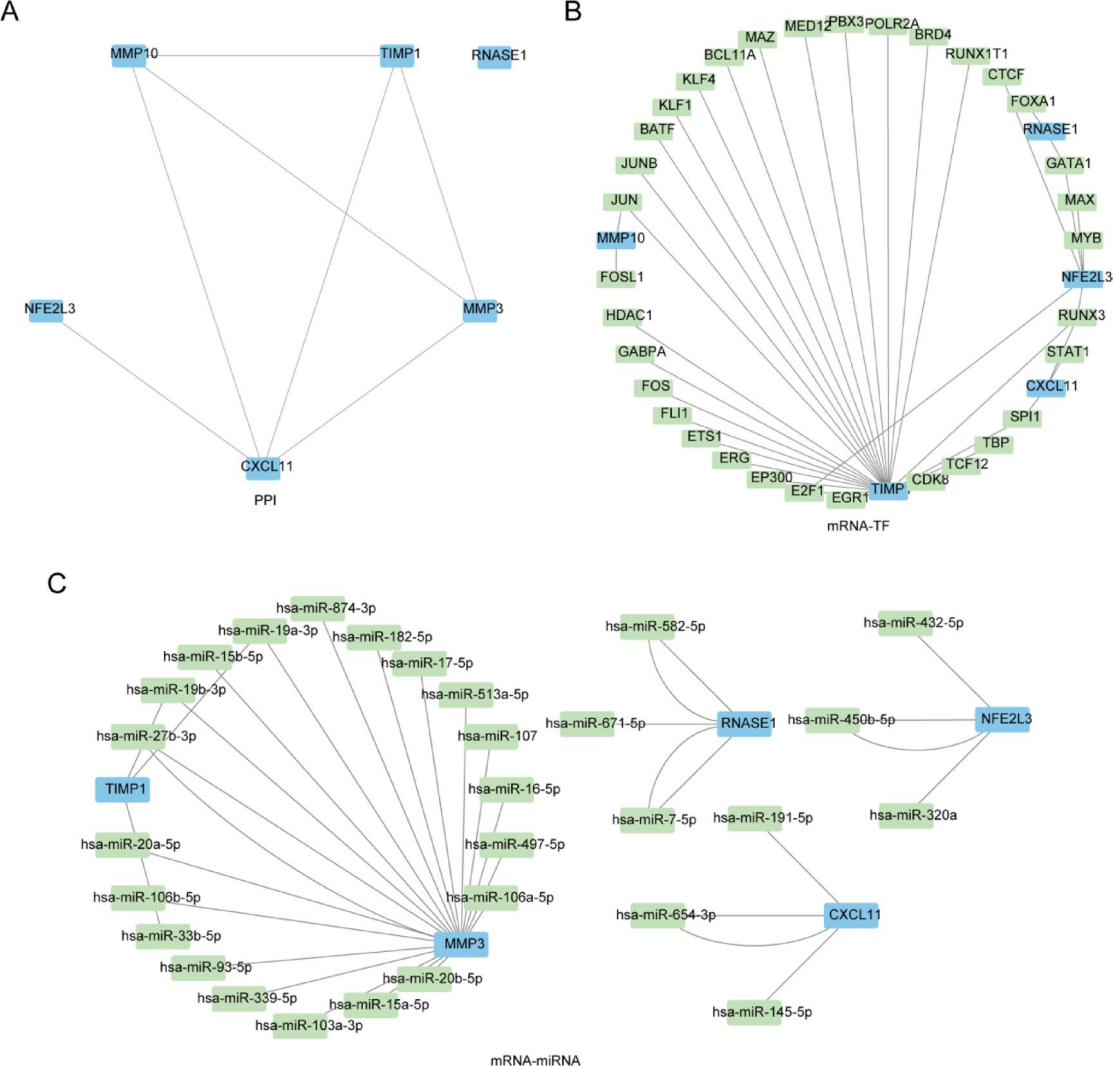
PPI network Of OXSRDEGs and mRNA-TF, mRNA-miRNA. (**A**) Illustrates the Protein–Protein Interaction (PPI) network of OXSRDEGs. (**B**) Showcases the mRNA-TF interaction network for OXSRDEGs, with blue quadrilaterals for mRNAs and cyan quadrilaterals for transcription factors (TFs). (**C**) Depicts the network between hub genes and miRNAs, using blue quadrilaterals for mRNAs and cyan for miRNAs. The PPI network indicates Protein–Protein Interaction; TF stands for Transcription Factors. OXSRDEGs are Oxidative Stress Related Differentially Expressed Genes.

Utilizing the STRING database, the protein–protein interaction (PPI) network of the 6 prognostic OXSRDEGs (*CXCL11, MMP10, MMP3, NFE2L3, RNASE1, TIMP1*) was analyzed, with the network visualized through Cytoscape software (Fig. 16A).

The study identified transcription factors (TFs) that bind to the 6 Prognostic OXSRDEGs using the CHIPBase database (version 3.0), uncovering 39 interactions between 5 mRNAs and 33 transcription factors (Table 7). This mRNA-TF interaction network was then depicted using Cytoscape software, with blue quadrilaterals representing mRNAs and cyan quadrilaterals denoting transcription factors (TFs) (Fig. 16B).

**Table 7:** mRNA-TF interaction network nodes.

mRNA	TF	mRNA	TF
CXCL11	RUNX3	TIMP1	GABPA
CXCL11	SPI1	TIMP1	HDAC1
CXCL11	STAT1	TIMP1	JUN
MMP10	JUN	TIMP1	JUNB
MMP10	FOSL1	TIMP1	BATF
NFE2L3	CTCF	TIMP1	KLF1
NFE2L3	E2F1	TIMP1	KLF4
NFE2L3	GATA1	TIMP1	BCL11A
NFE2L3	MAX	TIMP1	MAZ
NFE2L3	MYB	TIMP1	MED12
NFE2L3	RUNX3	TIMP1	PBX3
RNASE1	FOXA1	TIMP1	POLR2A
RNASE1	GATA1	TIMP1	BRD4
TIMP1	E2F1	TIMP1	RUNX1T1
TIMP1	EGR1	TIMP1	RUNX3
TIMP1	EP300	TIMP1	SPI1
TIMP1	ERG	TIMP1	TBP
TIMP1	ETS1	TIMP1	TCF12
TIMP1	FLI1	TIMP1	CDK8
TIMP1	FOS		

*TF* transcription factors.

Furthermore, mRNA-miRNA interaction predictions were conducted using data from the miRDB database for the 6 OXSRDEGs, focusing on interactions noted with “pancancerNum > 5”. The resulting mRNA-miRNA interaction network was visualized using Cytoscape software (Fig. 16C), where blue quadrilaterals symbolize mRNAs and cyan quadrilaterals represent miRNAs, comprising 5 mRNAs, 29 miRNAs, and 36 pairs of mRNA-miRNA interactions (detailed information in Table 8).

**Table 8:** mRNA-miRNA interaction network nodes.

miRNA	mRNA	miRNA	mRNA
hsa-miR-145-5p	CXCL11	hsa-miR-106b-5p	MMP3
hsa-miR-191-5p	CXCL11	hsa-miR-339-5p	MMP3
hsa-miR-654-3p	CXCL11	hsa-miR-20b-5p	MMP3
hsa-miR-654-3p	CXCL11	hsa-miR-497-5p	MMP3
hsa-miR-15a-5p	MMP3	hsa-miR-513a-5p	MMP3
hsa-miR-16-5p	MMP3	hsa-miR-874-3p	MMP3
hsa-miR-17-5p	MMP3	hsa-miR-320a	NFE2L3
hsa-miR-19a-3p	MMP3	hsa-miR-432-5p	NFE2L3
hsa-miR-19b-3p	MMP3	hsa-miR-450b-5p	NFE2L3
hsa-miR-20a-5p	MMP3	hsa-miR-450b-5p	NFE2L3
hsa-miR-93-5p	MMP3	hsa-miR-7-5p	RNASE1
hsa-miR-103a-3p	MMP3	hsa-miR-7-5p	RNASE1
hsa-miR-106a-5p	MMP3	hsa-miR-582-5p	RNASE1
hsa-miR-107	MMP3	hsa-miR-582-5p	RNASE1
hsa-miR-182-5p	MMP3	hsa-miR-671-5p	RNASE1
hsa-miR-15b-5p	MMP3	hsa-miR-19a-3p	TIMP1
hsa-miR-27b-3p	MMP3	hsa-miR-19b-3p	TIMP1
hsa-miR-27b-3p	MMP3	hsa-miR-33b-5p	TIMP1

## Discussion

CRC associated with ulcerative colitis (UC-CRC) often presents at advanced stages, leading to poorer outcomes.Oxidative stress plays a central role in both the pathogenesis of UC and CRC^4^. This study explores the interplay between ulcerative colitis, colorectal cancer, and oxidative stress. It employs differential expression analysis to identify Oxidative Stress Related Differentially Expressed Genes (OXSRDEGs) and conducts GO, KEGG, and GSEA analyses. The research also involves constructing Cox and LASSO models to predict patient prognosis, establishing PPI and mRNA-related networks to examine molecular interactions, and utilizing CIBERSORTx and ssGSEA algorithms for immune infiltration analysis.

Oxidative stress is a ubiquitous metabolic pathway in tumor cells. Gene Ontology (GO) analysis revealed that the genes related to Oxidative Stress Related Differentially Expressed Genes (OXSRDEGs) play a crucial role in the positive regulation of oxidative stress-induced cell death, neutrophil chemotaxis, and leukocyte migration. KEGG pathway analysis indicated that OXSRDEGs are involved in various inflammatory and chemokine signaling pathways, including the NF-kappa B and TNF signaling pathways, which are critical for tumor immunity and inflammatory responses.GSEA confirms enrichment in chemokine signaling pathways and inflammatory responses, highlighting the pro-inflammatory microenvironment’s contribution to cancer development. This pro-inflammatory microenvironment is a key contributor to cancer development.The *NFE2L3* transcription factor bridges NF-κB signaling to CDK1 activation, promoting the proliferation of colon cancer cells. Our research further confirmed the elevated expression of the OXSRDEGs *NFE2L3* in both CRC and UC across two datasets, with significant involvement in the NF-kB pathway. Its overexpression may lead to the continuous activation of the NF-kB pathway, IL-17’s interaction with inflammatory cells and signaling pathways suggests a role in immune suppression and colitis-associated colorectal cancer promotion^36–39^. Our findings corroborate that *MMP10* and *MMP3*, regulated by IL-17 and involved in the inflammation-cancer relationship, become upregulated, enhancing IL-17 signal transduction and the release of inflammatory mediators, thus facilitating the development of colitis-associated colorectal cancer^40^. The gradually changing expression levels of these genes may reflect their important regulatory role in colorectal cancer progression, suggesting that these genes may be potential disease markers and therapeutic targets.

Additionally, our findings suggest that Oxidative Stress Related Differentially Expressed Genes (OXSRDEGs) may encompass genes pivotal to immune regulation. Immune infiltration analysis revealed significant variances in most immune cell populations between diseased and control groups.*CXCL1*, *CXCL11*, *MGP*, and *PPARGC1A* exhibited a strong correlation with key immune cells such as Effector memory CD8 T cells, CD4 + T memory cells, M1 macrophages, and activated NK cells. Among the core genes identified, the chemokine *CXCL11* has been recognized for its pronounced pro-inflammatory effects in various autoimmune diseases^41^. Experimental studies have confirmed that *CXCL11* is significantly upregulated in both UC and CRC. miR-34a-5p and miR-203a-5p have been identified as potential regulators of *CXCL11*. Furthermore, *CXCL11* may mediate colitis-associated carcinogenesis (CAC) by activating the JAK-STAT signaling pathway through its interaction with cytokine receptors in UC^42^. Significant correlations were observed between *CXCL1* and CD4 + T memory cell infiltration, as well as between M1 macrophages and *CXCL11* infiltration, to some extent underscoring their diagnostic significance in CRC and UC. The strongest correlations were between Natural killer cells and *MGP*, and between Effector memory CD8 T cells and *PPARGC1A*, probably suggesting a synergistic interaction between immune cells and differential genes in disease pathogenesis. Our findings, supported by existing evidence, highlight the crucial role of OXSRDEGs related to immune cell infiltration in CRC and UC, warranting further experimental investigation.

Further validation of the ROC curve has revealed the diagnostic potential of several OXSRDEGs.A significant portion of the 12 OXSRDEGs displayed substantial diagnostic value for both diseases. Notably, the matrix metalloproteinase (*MMP*) family, includes *MMP9*, which has been extensively explored for its critical contribution to the invasion and metastasis of colorectal cancer. Decent research both domestically and internationally has revealed *MMP10*'s positive expression in the colon and rectum, correlating with the predissolution of type IV collagen in cancerous tissues^43^. This study also underscores the significant diagnostic capabilities of *MMP10* and *MMP3* in colorectal cancer. *PPARGC1A* has been reported to influence cell proliferation and invasion through the AKT/GSK-480β/β-catenin pathway in human CRC cells SW3 and SW620^44,45^. Moreover, Chen et al.^46^ identified the loss of *BMP5* as an early event in CRC, with low *BMP5* expression linked to recurrence and poor prognosis. This study uniquely demonstrates that *BMP5* and *PPARGC1A* not only correlate with the disease prognoses but also serve as effective diagnostic markers for both conditions.

OXSRDEGs may encompass genes crucial for the prognosis of ulcerative colitis and colorectal cancer, where their aberrant expression could be associated with tumor differentiation, metastasis risk, and patient survival.LASSO regression analysis identified six genes with prognostic significance, all exhibiting elevated expression in the disease group. Previous research indicates that elevated *TIMP1* expression significantly diminishes colorectal cancer patients’ overall survival rate, suggesting a poor prognosis^47^. Our findings also reveal, for the first time, that *TIMP-1*, classified among OXSRDEGs, is upregulated in both UC and CRC, with enrichment in tumor signaling pathways, such as TGF-β, probably marking it as a novel anticancer therapeutic target. *MGP*, known to facilitate CRC cell growth and proliferation by increasing intracellular calcium concentration and activating the NF-κB pathway^48^, contrasts with the downregulation of *CXCL11*, *CXCL2*, and *CXCL1* in CRC high-risk groups^49^. Our analysis indicated that the high-expression groups of *CXCL11*, *MMP10*, *MMP3*, *NFE2L3* are associated with better survival outcomes, whereas high expressions of *MGP*, *RNASE1*, *TIMP1* correlate with poorer survival, aligning with previous studies. Utilizing these genes, we developed a model to predict patient survival rates at 1, 3, and 5 years post-diagnosis, showcasing the model’s predictive accuracy for 5-year survival rates surpassing those for 3 and 1 year. The model can predict clinical prognosis to a certain extent.

We further established an mRNA regulatory network for the six prognostic Oxidative Stress Related Differentially Expressed Genes (OXSRDEGs), incorporating 29 miRNA molecules and generating 36 mRNA-miRNA interaction pairs. Key genes such as *CXCL11, NFE2L3*, and *RNASE1* are regulated by a multitude of miRNAs. Specifically, hsa-miR-191-5p, hsa-miR-654-3p, and hsa-miR-7-5p, all of which are miRNAs linked to tumorigenesis, have been identified to interact with *CXCL11*^50^. Additionally, hsa-miR-582-5p functions as a binding partner for lncRNA DCST1-AS1, which is known to modulate the invasiveness of colorectal cancer (CRC) cells via the hsa-miR-582-5p/HMGB1 pathway^51^. The expression of miR-671-5p is reduced in gastric cancer, and the inhibition of miR-671-5p has been shown to encourage the proliferation of gastric cancer cells^52^. These miRNAs also have the potential to interact with *RNASE1*. Concurrently, *TIMP1* is associated with various transcription factors, positioning it as a crucial gene and therapeutic target within the mRNA-TF interaction network research.

This study acknowledges several limitations: lack of experimental validation in a laboratory setting; Lack of clinical relevance studies, unable to integrate clinical data for analysis; The TCGA database does not specifically include data on inflammatory bowel disease (IBD) associated colorectal cancer (UC-CRC), so the suitability of selected oxidative stress-related genes in UC-CRC may be affected. The selection of data sets may result in batch variations or insufficient sample sizes, so larger cohorts are needed to ensure the reliability and stability of the study results. The results need to be further validated in more specific and diverse samples, and future studies should be combined with IBD-specific data to further explore the potential roles and mechanisms of these genes in UC-CRC.

## Electronic supplementary material

Below is the link to the electronic supplementary material.

Supplementary Material 1

## Data Availability

All data used in this study are derived from publicly available databases. The colorectal cancer (COADREAD) dataset and associated clinical data were obtained from The Cancer Genome Atlas (TCGA) using the TCGAbiolinks R package. More information about TCGA can be found at (https://www.cancer.gov/about/nci/organization/ccg/research/structural-genomics/tcga). The expression profile datasets GSE74602 and GSE4183 were accessed from the GEO database (https://www.ncbi.nlm.nih.gov/). Information on oxidative stress-related genes was retrieved from the GeneCards database.This study is authorized to allow Springer Nature Limited to publish under an open-access CC BY license.
