# Supplementary material for: Oxidative stress gene expression in ulcerative colitis: implications for colon cancer biomarker discovery
Source: Sci Rep. 2025 Jul 2;15:22641. doi: 10.1038/s41598-025-05108-8 (PMC12218319; doi:10.1038/s41598-025-05108-8)
Supplement: Supplementary file 1 — Supplementary Material 1 [file 41598_2025_5108_MOESM1_ESM.docx]

Oxidative Stress Gene as a Biomarker of Ulcerative Colitis-Associated Colon Cancer and Disease Pathogenesis

Ting Yan,Ting Su,Miaomiao Zhu3,Qiyuan Qing,Binjie Huang,Tenghui Ma

1. The time ROC of OXSRDEGs was negative.
